# Linking enzymatic hydrolysis to structural, volumetric, and hydrodynamic evolution of β–lactoglobulin in solution

**DOI:** 10.1016/j.fochx.2026.104152

**Published:** 2026-06-29

**Authors:** Mark Dizon

**Affiliations:** School of Chemistry, University College Dublin, Belfield, Dublin 4, Ireland; Department of Biomedical Science, Faculty of Health and Society, Malmö University, SE-205 06 Malmö, Sweden

**Keywords:** Protein hydrolysis, β–Lactoglobulin, Volumetric methods, Ultrasonic relaxation, Optical spectroscopy, Light scattering, Protein structural transition, Protein hydrodynamics

## Abstract

This paper presents an integrated real-time framework linking α–chymotrypsin hydrolysis of β–lactoglobulin to simultaneous changes in structural, volumetric and hydrodynamic properties in buffered media. High-resolution ultrasonic spectroscopy, complemented with densitometry, enabled real-time monitoring of peptide bond cleavage and changes in protein volume and compressibility. Multi-frequency ultrasonic relaxation analysis yielded molar adiabatic relaxation compressibility values of 2–4 × 10^−14^ m^3^ mol^−1^ Pa^−1^, previously not reported for the β–lactoglobulin system. Circular dichroism and intrinsic tryptophan fluorescence spectroscopy confirmed progressive disruption of secondary structure and exposure of the hydrophobic core, respectively. Light scattering method and viscometry revealed corresponding changes in hydrodynamic properties. Proteolysis in phosphate-buffered media produced a slight viscosity increase despite extensive fragmentation, consistent with an expansion from compact globules to coil-like fragments, potentially involving phosphate-protein interactions. This integrated approach offers a quantitative basis for characterizing enzymatic hydrolysis and guiding the design of functional protein ingredients.

## Highlights

• HR-US and densitometry correlate real-time progress of hydrolysis to volume changes.
• Volume analysis separates hydration, intrinsic structure, and relaxation contributions.
• Adiabatic relaxation compressibility in phosphate buffer: 2–4 × 10^−14^ m^3^ mol^−1^ Pa^−1^**.**
• Secondary and tertiary structures change in proportion to degree of hydrolysis.
• Slight viscosity increase in phosphate buffer despite reduction in molecular size.

## Graphical abstract

**Figure:**
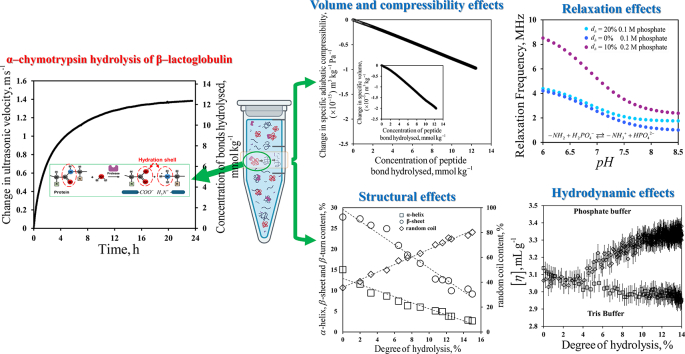

## 1 Introduction

Enzymatic hydrolysis is a widely used strategy to modify protein functional properties, enabling the tailored production of bioactive peptides for the food and pharmaceutical sectors (Cruz-Casas et al., 2021; Tang et al., 2023; Wu et al., 2020). These hydrolysates exhibit diverse health-promoting activities, including antihypertensive, antioxidative, and immunomodulatory effects, which are fundamentally linked to the resulting peptide sequences and their physical state in solution (Bielecka et al., 2022; Cruz-Casas et al., 2021; Dullius et al., 2018; Korhonen & Pihlanto, 2006). Among the most significant sources of these bioactive peptides are whey proteins, which have attracted extensive research interest due to their abundance, well-studied properties, high nutritional value and versatility in high-value food systems (Dullius et al., 2018; Olvera-Rosales et al., 2023).

Despite the industrial prevalence of proteolysis, a quantitative understanding of how peptide bond cleavage alters the physicochemical properties of proteins in solution remains incomplete. In particular, the relationship between the number of peptide bonds hydrolyzed, hydration, intrinsic structure, and hydrodynamic behavior is not yet fully resolved. Within this context, protein-water interaction plays a central role in determining macromolecular structure, stability, and solvation thermodynamics (Persson & Halle, 2018). Advances in analytical and computational biophysics have highlighted the intricate interrelationship between hydration dynamics and protein function (Durchschlag & Zipper, 2002; Linse & Hub, 2023). Upon solvation, proteins establish a complex hydration shell where water molecules form hydrogen bonds with surface polar groups, a phenomenon that can be assessed thermodynamically through volumetric analysis (Chalikian, 2003; Chalikian et al., 1996; Harding, 1997; Kharakoz & Sarvazyan, 1993; Lee et al., 2008) and structurally via X-ray or neutron scattering (Chalikian et al., 1996; Durchschlag & Zipper, 2002; Linse & Hub, 2023; Svergun et al., 1998). While such scattering methods provide atomic-level detail, they are often constrained by high costs and complex sample preparation. Alternatively, the combination of high-precision density and ultrasonic velocity measurements offers a fast, versatile, cost-effective, and highly sensitive means of probing the volumetric characteristics of protein solvation (Buckin, 2012; Dizon & Buckin, 2023; Papoutsidakis & Buckin, 2025).

The volumetric properties of proteins are particularly sensitive to volume change due to structural transition and hydration. Specific volume reflects the net change in hydration under constant conditions, while compressibility captures both hydration effects and internal atomic-level fluctuations (Chalikian et al., 1996; Kharakoz & Sarvazyan, 1993). The formation of a rigid hydration shell, driven by the electrostriction of water around newly exposed charged termini ($P_{1}-{\mathrm{COO}}^{-}$ and $P_{2}-{\mathrm{NH}}_{3}^{+}$), typically results in a significant negative change in compressibility, usually in aqueous solution of amino acids, small peptides and fully unfolded proteins which lack internal cavities (Kharakoz, 1997; Lee et al., 2008; Maya & Scanlon, 2020). In contrast, intact native and oligomeric proteins typically show positive contributions due to their soft packing structures and internal voids (Chalikian, 2003; Ochenduszko & Buckin, 2010). Enzymatic hydrolysis progressively exposes new charged groups and increases solvent accessibility, leading to enhanced hydration of the hydrolysate products and a corresponding decrease in compressibility of the reaction mixture (Maya & Scanlon, 2020). High-resolution ultrasonic spectroscopy (HR-US), combined with precision density measurements, enables real-time detection of these changes and has revealed a systematic decrease in compressibility and volume proportional to the extent of peptide bond hydrolysis (Buckin & Altas, 2017; Buckin & Craig, 2005; Dizon et al., 2020; Dizon & Buckin, 2023; Melikishvili et al., 2021; Papoutsidakis & Buckin, 2025). Such high-precision volume measurements provide detailed characterization of hydration changes associated with chemical reactions (Buckin & Altas, 2017), as well as accompanying secondary processes involving proton-transfer relaxations (Dizon and Buckin, 2023, Dizon and Buckin, 2025) and microstructural rearrangement (Ochenduszko & Buckin, 2010; Papoutsidakis & Buckin, 2025).

Complementary insights into the structural degradation of proteins during hydrolysis can be obtained using established optical spectroscopic techniques. Circular dichroism (CD) and Fourier-transform infrared (FT-IR) spectroscopy are widely used to monitor changes in secondary structure, including the transition from ordered (α–helix and β–sheet) to disordered conformations, and can be correlated with the degree of hydrolysis (Güler et al., 2011; Güler et al., 2016; Kristoffersen et al., 2020). Intrinsic tryptophan fluorescence has been used to probe the changes in protein tertiary structure and solvent exposure during proteolysis (Vorob'ev, 2020; Vorob'ev et al., 2011). The red-shift in tryptophan fluorescence spectra has been linked to the exposure of initially masked peptide bonds to solvent, i.e. demasking process, during protein unfolding and hydrolysis. This has been the basis of the quantitative two-step proteolysis model that distinguishes between demasking and hydrolysis kinetics and provides mechanistic insight into the structural dynamics of enzymatic protein degradation (Vorob'ev, 2025).

Hydrodynamic measurements provide precise estimates of size, shape, hydration, and molecular dimensions of proteins in solution (Durchschlag & Zipper, 2002; Zhou, 2001), and they have been used to yield insight into protein–solvent interactions and conformational dynamics in solution (García de la Torre & Hernández Cifre, 2020; Gonçalves et al., 2016; Harding, 1997). Dynamic light scattering (DLS) provides a powerful tool for studying diffusion behavior and calculating hydrodynamic radii from size and shape-dependent diffusion coefficients (Stetefeld et al., 2016). Viscometry, through measurement of intrinsic viscosity, has been extensively used to characterize the size and shape of proteins and when combined with other hydrodynamic methods, enables estimation of molecular parameters and shape factors (Karst et al., 2012). Despite their widespread application in polysaccharide and synthetic polymer science (Çatıker et al., 2000; Papoutsidakis & Buckin, 2023), such hydrodynamic characterization remains significantly underutilized in the context of protein hydrolysis (Su et al., 2008).

This work aims to investigate how volumetric, structural, and hydrodynamic properties evolve as a function of the extent of peptide bond hydrolysis during α–chymotrypsin digestion of β–lactoglobulin in buffered media. High-resolution ultrasonic spectroscopy and precision density measurements were used to quantify the degree of hydrolysis and the associated changes in volume and compressibility. The degree of peptide bond cleavage was then correlated with alterations in secondary and tertiary structure, as well as hydrodynamic properties. This integrated approach establishes a quantitative framework linking the degree of hydrolysis to hydration, conformational changes, and dilute solution hydrodynamics, providing new physicochemical insight into the solution-state behavior of proteins undergoing enzymatic transformation.

## 2 Materials and methods

### 2.1 Reagents and sample preparation

β–lactoglobulin from bovine milk (purity ≥90%, cat. L3908), α–chymotrypsin from bovine pancreas type VII (TLCK treated to inactivate residual trypsin activity, purity ≥90%, lyophilised powder, cat. C3142, with a declared enzyme activity of 64 units mg^−1^ protein) were purchased from Sigma Aldrich Co., Ltd. (Ireland). Potassium phosphate dibasic (purity ≥99%, cat. 60,353), potassium phosphate monobasic (purity ≥99.5%, cat. 60,218) and Tris(hydroxymethyl)aminomethane (ACS reagent, ≥ 99.8%, cat. 252,859) were purchased from Sigma Aldrich Co., Ltd. (Ireland). Ultrapure water (Millipore Super-Q-System, which includes a 0.22 μm membrane filter) with a resistivity of 18.2 MΩ.cm at 25 °C was used throughout.

All solids were weighed using an AG245 Mettler-Toledo microbalance (repeatability ± 0.02 mg). Buffer solutions (0.1 M phosphate or Tris) were freshly prepared before each experiment and adjusted to the target pH using a pre-calibrated Mettler-Toledo SevenCompact S220 pH meter equipped with an InLab® Expert Pro-ISM electrode (calibrated at pH 4.00, 7.00 and 9.21). β–lactoglobulin stock solutions (1.005% *w*/w) were prepared by dissolving the protein powder in buffer under gentle stirring overnight at room temperature to ensure complete dissolution. Minor pH deviations upon protein dissolution were corrected by incremental addition (2 μL aliquots) of dilute HCl or NaOH. α–Chymotrypsin stock solution (2.22% w/w) was freshly prepared in the same buffer immediately prior to use. Unless otherwise noted, 1.100 mL of protein substrate solution was degassed at room temperature and transferred to a sealed reaction cell. Enzymatic reaction was initiated by addition of 5 μL of enzyme solution using a Hamilton microsyringe, yielding final concentrations of 1.000% w/w β–lactoglobulin and 0.010% w/w α–chymotrypsin.

### 2.2 High-resolution ultrasonic spectroscopy

Ultrasonic velocity and attenuation during hydrolysis were measured using HR-US 102PT ultrasonic spectrometer (Sonas Technologies Ltd., Ireland) equipped with a precision programmable temperature controller (Julabo FS18) with temperature stability ± 0.01 °C. The measuring cell was filled with 1.100 mL of 1.005% (*w*/w) protein substrate solution, while the reference cell contained the corresponding buffer solution. Substrate and enzyme additions were performed using a pre-calibrated Hamilton syringe fitted with a precision-volume Chaney adapter. Following a 30-min equilibration period, 5.0 μL of freshly prepared α–chymotrypsin stock solution was injected into the measuring cell through a rubber septum.

Ultrasonic measurements were conducted in parallel at 2.9 MHz, 4.8 MHz, 7.9 MHz and 15.5 MHz. Reference values of ultrasonic velocity $u_{0}$ and ultrasonic attenuation $\alpha_{0}/f^{2}$ of pure water used at 25 °C were 1496.73 m s^−1^ and 2.19 × 10^−14^ s^2^ m^−1^, respectively. The evolution of ultrasonic velocity and attenuation was monitored continuously throughout the reaction.

The relative change in ultrasonic velocity, $\Delta u\equiv u-u_{0}$, per unit solute concentration $c_{i}$, under dilute and non-interacting conditions, is expressed as $a_{i}\;\left(\equiv \Delta u/u_{0}c_{i}\right)$, where u and $u_{0}$ the ultrasonic velocities of the solution and the solvent, respectively. The ultrasonic velocity data were scaled to zero minutes by extrapolating the first 2–3 min of the post-enzyme addition curve to time zero. The same procedure was applied to ultrasonic attenuation profiles. All measurements were repeated multiple times to ensure reproducibility.

### 2.3 Ultrasonic relaxation analysis

The frequency dependence of ultrasonic velocity and attenuation measured at 2.9, 4.8, 7.9 and 15.5 MHz was analyzed to characterize proton-transfer relaxation processes accompanying enzymatic hydrolysis in phosphate buffer. The analysis was performed using a dominant single-relaxation approximation, in which the observed relaxation was attributed primarily to proton exchange between phosphate ions and the newly formed N-terminal amino groups ($P_{2}-{{\mathrm{NH}}_{3}}^{+}\;$) of the protein hydrolysates:

(Reaction A) $$P_{2}-NH_{2}+H_{2}{PO_{4}}^{-}⇄P_{2}-{NH_{3}}^{+}+{HPO_{4}}^{2-}$$

The corresponding reaction-capacity equation (Eq. S4) is provided in Section D in supplementary material (SM).

The associated ultrasonic relaxation frequency $f_{\mathrm{rel}}$ was determined from the slope of the linear relationship between the frequency-dependent Δu and $\Delta \alpha /f^{2}$ (Eq. S5) at the four ultrasonic frequencies and representative stages of hydrolysis:

(1) $$\Delta u=\Delta u_{\infty }-f_{rel}\frac{u^{2}}{2\pi }\left(1+\psi_{ua}\right)\frac{\Delta \alpha }{f^{2}}$$

where $\Delta u_{\infty }$ is the ultrasonic velocity increment at the high-frequency limit and $\psi_{\mathrm{ua}}$ is a small concentration-dependent correction term. Subsequently, the effective forward proton-transfer rate constant $k_{f}^{0}$ was determined from the dependence of $f_{\mathrm{rel}}$ on the concentration factor of the reacting species, $\left(c_{H_{2}PO_{4}^{-}T}\left(x_{H_{2}PO_{4}^{-}}/x_{NH_{3}^{+}}\right)+c_{NH_{3}^{+}T}\left(1-x_{NH_{3}^{+}}/1-x_{H_{2}PO_{4}^{-}}\right)\right)$ (Eq. S6). The adiabatic reaction volume $∆V_{S}$ associated with the proton-transfer equilibrium was determined by simultaneous fitting of attenuation data measured at all four ultrasonic frequencies using the single-relaxation model (Eq. S7). Brief summary of the relaxation model, parameter definitions and fitting procedures is provided in the Supplementary Information.

### 2.4 Determination of concentration of peptide bonds from ultrasonic measurements

Under dilute and non-interacting conditions, the change in ultrasonic velocity in the reaction mixture, $\delta u\left(t\right)$, during time interval δt at reaction time t, is linked with the change in concentration of bonds hydrolyzed $\delta c_{bh}\left(t\right)$, within the same time interval by (Dizon & Buckin, 2023):

(2) $$\delta c_{\mathrm{bh}}\left(t\right)=\frac{\mathrm{\delta u}\left(t\right)}{u_{0}\Delta a_{h}\left(t\right)}$$

where $\Delta a_{h}$ is the concentration increment of ultrasonic velocity associated with the peptide bond hydrolysis. The calculation algorithm applied iterative numerical integration of the measured ultrasonic velocity profiles with explicit correction for ionization equilibria associated with the newly generated terminal amino and carboxyl groups, titratable amino acid side chains, and buffer species present in the reaction mixture. The full iterative expression is:

(3) $$c_{bh}\left(t_{n+1}\right)=c_{bh}\left(t_{n}\right)+\frac{u\left(t_{n+1}\right)-u\left(t_{n}\right)}{u_{0}\Delta a_{h}\left(t_{n}\right)}$$

where symbol and the subscript $\left(t_{n+1}\right)$ and $\left(t_{n}\right)$ characterize the times at which the parameters are taken, and the parameter $\Delta a_{h}\left(t_{n}\right)$ is calculated according to Eqs. (4) and (5)

(4) $$\Delta a_{h}=\Delta a_{P}+\Delta a_{I}\;\Delta a_{I}=\Delta a_{COOH}x_{COOH}-\Delta a_{NH_{3}^{+}}\left(1-x_{NH_{3}^{+}}\right)+\left(1-x_{NH_{3}^{+}}-x_{COOH}\right)\frac{G}{D}\;G=\sum_{i=1}^{k}\Delta a_{AH_{i}}c_{AHT_{i}}x{'}_{AH_{i}}-\Delta a_{H_{2}O}\gamma_{H^{+}}10^{\left(pH-pK_{w}\right)}\times \ln\left(10\right)\left(\frac{1\;0^{3}mol\;m^{-3}}{\rho }\right)\;D=\sum_{i=1}^{k}c_{AHT_{i}}x{'}_{AH_{i}}-\left(\frac{1}{\gamma_{H^{+}}}10^{-pH}+\gamma_{H^{+}}10^{\left(pH-pK_{w}\right)}\;\right)\times \ln\left(10\right)\;\left(\frac{1\;0^{3}mol\;m^{-3}}{\rho }\right)\;\;$$

where $\Delta a_{P}$ and $∆a_{I}$ are the change of concentration increment of ultrasonic velocity due to the protein hydrolysis and ionization; $∆a_{\mathrm{COOH}}$, $\Delta a_{{\mathrm{NH}}_{3}^{+}}$ and $\Delta a_{\mathrm{AH}\;i}$ are the change of concentration increment of ultrasonic velocity due to the protonation of $P_{2}-{\mathrm{NH}}_{3}^{+}$ and $P_{1}-\mathrm{COOH}$, and other titratable atomic groups present in the reaction mixture, AH**,** i; $x_{NH_{3}^{+}}$ $\left(\equiv N_{{\mathrm{NH}}_{3}^{+}}/\left(N_{{\mathrm{NH}}_{2}}+N_{{\mathrm{NH}}_{3}^{+}}\right)\right)$ and $x_{\mathrm{COOH}}$ $\left(\equiv N_{\mathrm{COOH}}/\left(N_{\mathrm{COOH}}+N_{{\mathrm{COO}}^{-}}\right)\right)$ where N is the number of moles, are the fraction of the protonated form of $P_{2}-{\mathrm{NH}}_{3}^{+}$ and $P_{1}-\mathrm{COOH}$, respectively; $x_{{\mathrm{AH}}_{i}}$ is the fraction of protonated form of the weak acid AH**,** i; $c_{AHT_{i}}$ is the total concentration of the other titratable groups i protonated and deprotonated; $\gamma_{H^{+}}$ is the activity coefficient of hydrogen ion $H^{+}$; and ρ is the density of the solution. Under the absence of specific interactions, the differentiation of $x_{\mathrm{AH}\;i}$ with respect to pH provides

(5) $$x{'}_{AH_{i}}=\ln\left(10\right)x_{AH_{i}}\left(x_{AH_{i}}-1\right)$$

The values of concentration increments of ionization of relevant functional groups, $\Delta a_{\mathrm{AHi}}$, and ${\mathrm{pK}}_{A\;i}^{\mathrm{app}}$ required for such calculations were discussed previously and are given in Table S1. Detailed derivations and fitting algorithms are also provided in the previous work (Dizon & Buckin, 2023).

The resulting $c_{\mathrm{bh}}\left(t\right)$ profiles were converted to the time profile of degree of hydrolysis $d_{h}\left(t\right)$, according to:

(6) $$d_{h}\left(t\right)=\frac{\delta c_{\mathrm{bh}}\left(t\right)}{{c_{b}}^{0}}$$

where ${c_{b}}^{0}$ is the initial concentration of hydrolysable peptide bonds in the protein substrate. Additionally, assuming random cleavage of a linear polymer chain, the number-average molar mass, $\bar{M_{n}}$ was calculated from the measured real-time hydrolysis profiles as:

(7) $$\bar{M_{n}}=\frac{{M_{P}}^{0}-M_{H_{2}O}}{1+c_{bh}\frac{{M_{P}}^{0}}{w^{0}}}+M_{H_{2}O}$$

where ${M_{P}}^{0}$ is the initial molar mass of proteins, in kg mol^−1^, $w^{0}$ (in kg kg^−1^ of mixture) is the initial weight fraction of the protein, and $M_{H_{2}O}$ (= 0.018 kg mol^−1^) is the molar mass of water.

### 2.5 TNBS assay for independent determination of degree of hydrolysis

*TNBSA assay*. The degree of hydrolysis was determined independently by the 2,4,6-trinitrobenzenesulfonic acid (TNBS) colorimetric assay following the procedure of Dizon and Buckin (Dizon & Buckin, 2023). Hydrolysate samples were prepared in parallel with ultrasonic measurements. Briefly, 2.20 mL of protein substrate solution (1.005% *w*/w, pre-filtered through 0.1 μm syringe filters) was dispensed into reaction vials using a Hamilton syringe with a fixed-volume adapter and pre-equilibrated at 25 °C for 30 min. Hydrolysis was initiated by addition of 10.0 μL of enzyme solution, after which the mixture was inverted for 30 s and incubated at 25 °C. At defined time intervals, 100 μL aliquots were withdrawn and quenched by addition of 25.0 μL of 1 M HCl to inactivate the enzyme. Quenched samples were sealed and stored at −25 °C until analysis. For the colorimetric reaction, duplicate aliquots of 30.0 μL of each acidified sample were diluted into 970 μL of 0.17% (*w*/*v*) sodium dodecyl sulfate (SDS; BioReagent, ≥98.5% GC, L3771, Sigma-Aldrich, Germany). The corresponding blank samples were prepared with 1.000 mL of the appropriate concentration of SDS solution without the analyte. Each sample and blank was further diluted sequentially with 1.000 mL of 0.21 mol L^−1^ sodium phosphate buffer (pH 8.2), followed by 1.000 mL of 0.03% (w/v) TNBS solution (BioReagent, 5% w/v stock in H₂O, P2297, Sigma-Aldrich, Germany). Mixtures were vortexed immediately and incubated at 50 °C for 60 min away from light. The reaction was terminated by addition of 2.000 mL of 0.100 mol L^−1^ HCl, giving a total reaction volume of 5.000 mL. Samples were cooled down, away from light, for 30 min and absorbance was recorded at 340 nm using a Cary 60 UV–Visible spectrophotometer (Agilent Technologies, USA) at 25 °C. A previously established leucine calibration curve gave a molar extinction coefficient of $\varepsilon_{340}$ = 11,507 ± 278 M^−1^ cm^−1^.

TNBSA assay quantifies the free primary amino groups generated during enzymatic hydrolysis. Because each peptide bond cleavage produces one new $P_{2}-{NH_{3}}^{+}\;$ group, its concentration is equal to the concentration of hydrolyzed peptide bonds $c_{bh}$ which can be quantified as:

(8) $$c_{bh}\left(t\right)=\left[A_{N}\left(t\right)-A_{N}^{0}\right]\times 518.75$$

where $A_{N}\left(t\right)$ and $A_{N}^{0}$ are the N-terminal amino group concentrations determined from the measured absorbance at 340 nm using $\varepsilon_{340}$ at time t and time zero of hydrolysis. The factor 518.75 accounts for the overall dilution introduced during sample preparation and TNBS analysis.

*pH method*. For hydrolysis reactions conducted in 0.1 M Tris buffer at pH 7.8, the concentration of hydrolyzed peptide bonds was independently determined from continuous pH monitoring method with precision of ± 0.003. A Mettler-Toledo SevenCompact S220 pH meter with an InLab® Expert Pro-ISM microelectrode (calibrated at 4.00, 7.00 and 9.21) was used. The initial pH of the protein substrate before enzyme addition was recorded. The pH evolution during hydrolysis was monitored continuously at a sampling rate of one measurement every 2 min throughout the reaction.

To account for instrumental pH drift, the pH of 0.1 M Tris buffer without protein or enzyme was measured under the same duration and conditions across multiple independent runs; the averaged pH drift profile, $pH_{ref}\left(t\right)$, was used to correct the measured hydrolysis pH, $pH_{measured}\left(t\right)$, according to ${\mathrm{pH}}_{\mathrm{corrected}}\left(t\right)={\mathrm{pH}}_{\mathrm{measured}}\left(t\right)-{\mathrm{pH}}_{\mathrm{ref}}\left(t\right)$. The time profiles of ${\mathrm{pH}}_{\mathrm{corr}}\left(t\right)$ was then used to calculate the concentration of peptide bonds hydrolyzed iteratively from the initial pH value at time zero:

(9) $$\left(c_{bh}\left(t_{n+1}\right)-c_{bh}\left(t_{n}\right)\right)=\left(pH_{corr}\left(t_{n+1}\right)-pH_{corr}\left(t_{n}\right)\right)\frac{D\left(t_{n}\right)}{1-x_{NH_{3}^{+}}\left(t_{n}\right)-x_{COOH}\left(t_{n}\right)}$$

where the parameter D given in Eq. (4), as well as parameters $x_{i}$ and $\;x{'}_{i}$ in Eq. (5); $\;c_{{\mathrm{NH}}_{3}^{+}\;T}\left(t_{n}\right)=c_{\mathrm{COOH}\;T}\left(t_{n}\right)=c_{\mathrm{bh}}\left(t_{n}\right)+c_{{\mathrm{NH}}_{3}^{+}\;T}^{0}$, where $c_{{\mathrm{NH}}_{3}^{+}\;T}^{0}$ and $\;c_{NH_{3}^{+}\;T}\left(t_{n}\right)$ are consequently the concentration of terminal amino (and carboxyl) groups in mol kg^−1^ at reaction time zero and time $t_{n}$. In the case of hydrolysis of individual ‘native’ proteins, $c_{{\mathrm{NH}}_{3}^{+}\;T}^{0}$ is equal to the protein concentration at reaction time zero. A more detailed description of the calculation algorithm can also be found in the previous publication (Dizon & Buckin, 2023).

### 2.6 Density measurement and volume analyses

Change in solution density during protein hydrolysis was measured using a vibrating-tube densitometer (DMA 5000 M, Anton Paar) with temperature stability ± 0.001 °C and a density resolution of 0.000001 g·cm^−3^. Hydrolysis was initiated by adding 5.0 μL of 2.22% (*w*/w) α–chymotrypsin solution to 1.100 mL of pre-degassed 1.005% (w/w) protein substrate solution. The reaction mixture was briefly vortexed, transferred into a syringe, and injected into the densitometer's U-tube inlet. Following a 2 min equilibration period, density measurements were recorded continuously using custom Python-based acquisition software.

The measured density profile $\rho \left(t\right)$ was converted to specific volume $v\left(t\right)=1/\rho \left(t\right)$. The time-dependent change in specific volume $\mathrm{\delta v}\left(t\right)$ and specific adiabatic compressibility $\delta k_{S}\left(t\right)$ were evaluated from the combined density and ultrasonic velocity data using the Newton–Laplace relation, i.e. $u=v/\sqrt{k_{S}}$:

(10) $$\mathrm{\delta v}\left(t\right)=v_{t}-v^{0}$$

(11) $$\delta k_{S}\left(t\right)={\left(\frac{v^{0}}{u^{0}}\right)}^{2}\frac{2\left(\frac{\mathrm{\delta v}\left(t\right)}{v^{0}}-\frac{\mathrm{\delta u}\left(t\right)}{u^{0}}\right)+{\left(\frac{\mathrm{\delta v}\left(t\right)}{v^{0}}\right)}^{2}-{\left(\frac{\mathrm{\delta u}\left(t\right)}{u^{0}}\right)}^{2}}{{\left(1+\frac{\mathrm{\delta u}\left(t\right)}{u^{0}}\right)}^{2}}$$

where $v_{t}\;$ and $v^{0}$ are the specific volumes at time t and time 0, respectively.

### 2.7 Circular dichroism spectroscopy

Secondary structural changes of the protein during hydrolysis were monitored by far-UV circular dichroism (CD) spectroscopy. A reaction vial containing 1.100 mL of 1.005% (*w*/w) β–lactoglobulin solution, previously filtered through a 0.1 μm syringe filter, was pre-equilibrated at 25 °C for 30 min using a Thermo HAAKE C25P refrigerated bath with Phoenix Controller. Hydrolysis was initiated by adding 5.0 μL of 2.22% (w/w) α–chymotrypsin solution using a Hamilton precision syringe. The mixture was immediately stirred by gentle inversion for 30 s and incubated at 25 °C. At selected time points, 10.0 μL aliquots were withdrawn from the reaction mixture and immediately quenched in 990.0 μL Bowman–Birk inhibitor (0.02 w/w% in 0.1 M phosphate buffer pH 7.8) solution. The quenched samples were vortexed and transferred into quartz CD cuvettes for analysis. CD spectra were recorded on a JASCO J-810 spectropolarimeter over the wavelength range 260 nm to 185 nm.

The raw ellipticity (degrees) was baseline-corrected against a blank containing the enzyme and Bowman–Birk inhibitor in buffer, then converted to mean residue ellipticity ${\left[\theta \right]}_{MRW}$ (in deg. cm^2^ dmol^−1^) using:

(12) $${\left[\theta \right]}_{\mathrm{MRW}}=\frac{\mathrm{MRW}\times \theta_{\mathrm{corr}}}{10\times l\times C}$$

where $\theta_{\mathrm{corr}}$ is the corrected ellipticity in degrees unit after baseline correction, l (= 0.1 cm) is the pathlength of the cuvette, C is the total concentration of the protein (intact plus hydrolysis products) and after the addition of the inhibitor in g mL^−1^ unit, and MRW is the mean residue weight calculated as: $MRW={M_{P}}^{0}/\left(N_{aa}-1\right)$ where ${M_{P}}^{0}$ is the initial molecular mass of the protein and $N_{\mathrm{aa}}$ is the number of amino acid in the primary sequence of the proteins. For β–lactoglobulin, MRW ∼ 113.7 g mol^−1^, taking ${M_{P}}^{0}$ ∼ 18,300 g mol^−1^ and $N_{\mathrm{aa}}$ = 162. Contributions from α–chymotrypsin were negligible given the 100:1 substrate-to-enzyme mass ratio.

Secondary structure analysis was performed using CDPro, which integrates multiple algorithms and reference datasets for protein CD spectral interpretation. In this study, three established methods: SELCON3, CONTINLL, and CDSSTR, were employed (Güler et al., 2016; Sreerama et al., 2000; Sreerama & Woody, 2000). The SP175 reference set, optimized for the 190–240 nm range, was used for all calculations (Micsonai et al., 2015). The quality of each fit was evaluated using the normalized root-mean-square deviation (NRMSD) as a goodness-of-fit metric. For each spectrum (190–240 nm), six structural elements were estimated: regular α–helix (H(r)), distorted α–helix (H(d)), regular β–sheet (S(r)), distorted β–sheet (S(d)), β–turns (T), and unordered structures (i.e. extended conformation) (U).

### 2.8 Intrinsic tryptophan fluorescence spectroscopy

Intrinsic fluorescence spectra were recorded using a Lambda LB50 luminescence spectrometer (PerkinElmer, Rodgau, Germany). A total of 3.300 mL of 1.005% (*w*/w) β–lactoglobulin substrate solution was transferred into a quartz fluorescence cuvette (1 cm pathlength), and baseline fluorescence spectra were recorded prior to initiation of hydrolysis. Hydrolysis was initiated by injection of 15.0 μL of 2.22% (w/w) α–chymotrypsin solution into the cuvette. The sample was rapidly mixed and returned to the thermostated measurement position. Fluorescence emission spectra were acquired using an excitation wavelength of 295 nm, with excitation and emission bandwidths set to 5 nm. Excitation at 295 nm was chosen to preferentially excite tryptophan over tyrosine residues. Emission spectra were recorded over 250–390 nm at a scan speed of 400 nm min^−1^. Background fluorescence from the buffer was subtracted from all spectra.

The apparent shift in fluorescence emission maximum was analyzed using the normalized masking relationship proposed by Vorob'ev et al. (Vorob'ev, 2022; Vorob'ev et al., 2011). The emission maximum wavelength $\lambda_{\max}$ at each time point was determined by fitting the fluorescence spectrum within a ± 15–25 nm window around the spectral peak to a quadratic function $F\left(\lambda \right)=a\lambda^{2}+b\lambda +c$, from which $\lambda_{\max}=-b/2a$. The degree of demasking was calculated as:

(13) $$1-x\left(t\right)=\frac{\lambda_{\max}\left(t\right)-\lambda_{\max}^{0}}{\lambda_{\max}^{\infty }-\lambda_{\max}^{0}}$$

where $x\left(t\right)$ represents the fraction of masked (native-like) state, $\lambda_{\max}\left(t\right)$ is the changes in the emission maximum wavelength at hydrolysis time t, $\lambda_{max}^{0}$ is the initial maximum emission wavelength at t = 0, and $\lambda_{max}^{\infty }$ corresponds to the limiting emission maximum wavelength at extensive hydrolysis.

### 2.9 Light scattering measurement

The evolution of intensity-weighted mean hydrodynamic diameter $Z_{Ave}$ and light scattering intensity I during enzymatic hydrolysis were monitored using dynamic light scattering (DLS) method on a photon correlation spectrometer (Malvern Mastersizer, Malvern Ltd., UK). Measurements were performed at a fixed scattering angle of 90°, using an argon laser (100 mW) operating at a wavelength of $\lambda_{0}$ = 488 nm. The temperature was maintained at 25.00 ± 0.01 °C with a Julabo thermostatic water bath.

Prior to the measurement, β–lactoglobulin stock solution (1.005%, *w*/w) was filtered sequentially through 0.10 μm and 0.02 μm syringe filters into a clean, dust-free round bottom quartz cuvette (1 cm diameter) to a final volume of 1.100 cm^3^. Protein loss after filtration was estimated to be below 1% based on relative UV absorbance measurements. The Rayleigh ratio of toluene was measured before each measurement session for instrument standardization. Hydrolysis was initiated by injection of 5.0 μL of freshly prepared α–chymotrypsin solution using a Hamilton precision syringe, and the time evolution of scattering intensity and hydrodynamic size was monitored continuously.

Data acquisition and analysis were performed using the Malvern Automeasure software (DTS v1.61, Malvern Ltd., UK). Each intensity autocorrelation function was collected from three consecutive acquisitions of 60 s duration, analyzed by the cumulant method to obtain intensity-weighted mean hydrodynamic diameter, $Z_{Ave}$ and polydispersity index. The correlator was operated in parallel mode with 64 channels arranged into four subgroups of progressively increasing sampling times. The shortest sampling time was 0.2 μs, with a dilation factor of 4, giving subsequent sampling times of 0.8, 3.2, and 12.8 μs. Delay times were extended to approximately 3.5 times the longest relaxation time observed to ensure complete characterization of the relaxation behavior of both the native protein and its hydrolysis products. All measurements were performed in duplicate, and average values are reported.

Apparent weight-average molecular mass $\bar{M_{W}}$ was calculated from the measured intensity of scattering data $I_{P}$ using the Rayleigh equation (Eq. S8) adapted for small globular proteins with given particle diameter much smaller than the laser wavelength $\lambda_{0}$= 488 nm, so that the angular factor $P\left(\theta \right)$ ≈ 1:

(14) $$\bar{M_{W}}=\frac{1}{\frac{\mathrm{KC}}{R_{\theta }}-2A_{2}C}$$

where K is the optical constant is given by Eq. S9, C is the total concentration of the protein (intact plus hydrolysis products) in g mL^−1^ unit, $R_{\theta }$ is the Rayleigh ratio (calculated relative to toluene, using its refractive index and Rayleigh ratio at 488 nm) is given by Eq. S10, and $A_{2}$ is the second virial coefficient. Given the dilute protein concentration (1% *w*/w) and small protein particle size, $A_{2}$ values were equal to 0.0 cm^3^ mol g^−1^ for measurement at pH 7.8 (monomeric protein), and equal to 1.6 × 10^−4^ cm^3^ mol g^−1^ for measurement at pH 7.0 (dimeric protein) (Baussay et al., 2004).

### 2.10 Rolling-ball viscometry

Viscosity measurements were carried out using a rolling-ball microviscometer (Lovis 2000 M/ME, Anton Paar GmbH, Germany) equipped with a gold capillary. Instrument calibration was conducted according to the manufacturer's instructions, using distilled water at 25 °C, including air/water and density verification adjustment. The hydrolysis reaction mixture, containing 1% w/w β–lactoglobulin and 0.01% w/w α–chymotrypsin, was pre-activated in a thermostated vial at 25 °C before it was carefully injected into the glass capillary (1.59 mm inner diameter) via Teflon tubing, avoiding bubble formation. Temperature was maintained at 25 °C with an accuracy of ± 0.02 °C. Dynamic viscosity, kinematic viscosity, shear rate, Lovis Variation Coefficient, and Lovis Fw/Bw Deviation were recorded during each experiment. All measurements were performed in triplicate, and average values are reported and used in subsequent calculations.

Intrinsic viscosity $\left[\eta \right]$ was estimated from the measured dynamic viscosity η at a single protein concentration (5 mg mL^−1^, total protein plus hydrolysis products) using the Solomon-Ciutǎ single-point approximation (Solomon & Ciutǎ, 1962):

(15) η=2ηsp−lnηrel12C

where $\eta_{\mathrm{rel}}=\eta /\eta_{0}$ is the relative viscosity of the protein mixture to solvent; and $\eta_{\mathrm{sp}}=\eta_{\mathrm{rel}}-1$ is the specific viscosity. This method yields [η] with a typical error below 5%.

### 2.11 Statistical analysis

All measurements of α–chymotrypsin hydrolysis of β–lactoglobulin were performed in at least duplicate. Results are reported as mean ± standard error. Differences between experimental conditions, including buffer type (phosphate versus Tris) and pH (7.0 versus 7.8), were evaluated using analysis of variance (ANOVA), with statistical significance accepted at *p* < 0.05. Data processing, calculations, and graphical presentation were performed using Microsoft Excel, whereas statistical analyses were conducted using MATLAB R2024b (MathWorks, Natick, MA, USA).

## 3 Results and discussion

### 3.1 Volume and compressibility profiles

**Fig. 1:**
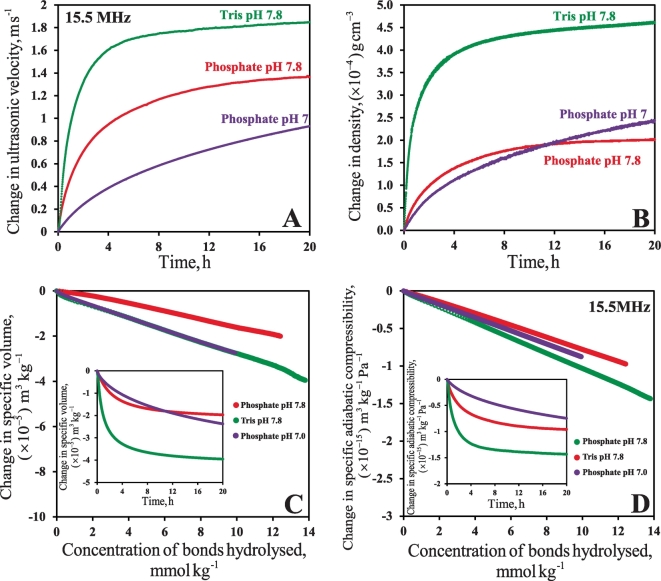
Change in ultrasonic velocity (A) at 15.5 MHz and density (B), and the Change in specific volume Δv (C) and in specific adiabatic compressibility $\Delta k_{S}$ (D) profiles during the α–chymotrypsin-catalyzed hydrolysis of β–lactoglobulin. The hydrolysis reaction was carried out at an enzyme concentration of 0.01% w/w and at a protein concentration of 1% w/w in 0.1 M phosphate buffer pH 7.0 (purple symbols) and 7.8 (red symbols), and Tris buffer pH 7.8 (green symbols) at 25 °C. (For interpretation of the references to colour in this figure legend, the reader is referred to the web version of this article.)

Fig. 1A shows the time-dependent change in ultrasonic velocity at 15.5 MHz during enzymatic hydrolysis of β–lactoglobulin (1% *w*/w) by α–chymotrypsin (0.01% w/w) at 25 °C in three different reaction media – 0.1 M phosphate buffer pH 7.8 and pH 7.0, and 0.1 M Tris buffer pH 7.8. All the presented profiles exhibit an increase in ultrasonic velocity which is related to the decrease in the adiabatic compressibility $k_{S}=1/\left(\rho u^{2}\right)$ of the reaction mixture (calculated from measured velocity and density data via the Newton–Laplace relation, as described in Eqs. (10) and (11)). This decrease in specific compressibility is exerted by the increased hydration of structural motifs and functional groups in the protein hydrolysate products compared to the parent β–lactoglobulin. Particularly, as peptide bonds are cleaved, newly exposed charged termini ($P_{1}-{\mathrm{COO}}^{-}$ and $P_{2}-{{\mathrm{NH}}_{3}}^{+}\;$) interact strongly with the water, forming rigid hydration shell that is significantly less compressible than bulk water, consequently decreasing the overall specific compressibility of the mixture, consequently increasing the ultrasonic velocity. The magnitude of this effect depends on the degree of ionization of the terminal groups and is, therefore, sensitive to pH and buffer composition.

At 15.5 MHz, where ultrasonic relaxation contributions from proton-transfer equilibria are negligible (see discussions in section 3.2 and (Dizon & Buckin, 2025)), the evolution of Δu is directly proportional to the concentration of hydrolyzed peptide bonds $c_{bh}\left(t\right)$ (Eq. (2), expressed in mol kg^−1^ protein mixture) (Dizon & Buckin, 2023). The proportionality coefficient to this relationship is expressed by $\Delta a_{h}\left(\delta pH\left(\delta c_{bh}\right)\right)=\Delta a_{P}+\Delta a_{I}\left(\delta pH\left(\delta c_{bh}\right)\right)$, where $\Delta a_{P}$ represents the intrinsic contribution of peptide bond cleavage, arising primarily from changes in hydration and compressibility of the polypeptide backbone. Previously reported values of $\Delta a_{P}$ for whey protein hydrolysis are 0.095 ± 0.0035 kg mol^−1^ and 0.090 ± 0.005 kg mol^−1^ for enzymatic hydrolysis of whey proteins at pH 7 and 7.8, respectively, (Dizon & Buckin, 2023). The term $\Delta a_{I}$ accounts for secondary ionization effects and is evaluated as function of pH change accompanying the increase in $c_{bh}\left(t\right)$ (Eq. (4)) during hydrolysis. This proportionality is valid under dilute solution conditions where specific interactions between reactants and products, as well as protein aggregation or self-association, are negligible.

During hydrolysis, the acidity of the medium increases as newly generated terminal carboxyl groups undergoes complete deprotonation and terminal amino groups become partially deprotonated. The resulting decrease in pH shifts the ionization equilibria of the newly formed termini and all other titratable species present (phosphate, amino acid side chains), each contributing distinct hydration and compressibility effects to $\Delta a_{I}$. These contributions are quantified via Eqs. (4) and (5), using the $pK_{A}$ values and concentration increment of ionization parameters listed in Table S1. Because $\Delta a_{P}$ is constant throughout the hydrolysis, the shift in $\Delta a_{h}$ arises solely from changes in $\Delta a_{I}$. This change is more pronounced at pH 7.8, where the relatively weak buffering capacity allows larger pH, whereas at pH7, the stronger buffer capacity renders the corresponding shift negligibly small. In contrast, $\Delta a_{I}$ in Tris buffer is negligibly small relative to $\Delta a_{P}$ under the hydrolysis condition performed and therefore its contribution can be neglected for practical purposes. Consequently, the measured $\Delta a_{h}$ in Tris directly approximates $\Delta a_{P}$ without requiring ionization correction.

In parallel, the change in solution density Δρ was measured in real-time during the hydrolysis (Fig. 1B). The density profile follows the same trend as in ultrasonic velocity as both probe changes in hydration and protein structures in solution (Lee et al., 2008; Taulier & Chalikian, 2001). Since density is inversely related to specific volume, the progressive increase in density indicates a net negative volume effect resulting from peptide bond cleavage and the subsequent increased hydration of the resulting products. Combining the measured density and ultrasonic velocity data through the Newton–Laplace relation enables quantitative determination of changes in specific volume $\mathrm{\delta v}\left(t\right)$ (Eq. (10) derived from v=1/ρ) and specific adiabatic compressibility $\delta k_{S}\left(t\right)$ (Eq. (11) derived from $k_{S}=1/\rho u^{2}$) in hydrolysis mixture, as shown in the insets of Fig. 1C and D, respectively.

The apparent changes in molar volume $\Delta V_{h}$ and adiabatic compressibility $\Delta K_{Sh}$ associated with hydrolysis were extracted by relating the time-dependent profiles of $\delta v\left(t\right)$ and $\delta k_{S}\left(t\right)$ profiles (insets of Fig. 1C and D, respectively) to the corresponding ultrasonically determined $c_{\mathrm{bh}}\left(t\right)$. Assuming that thermodynamic contributions per peptide bond cleavage are additive and approximately constant under dilute solution conditions, the slope of the corresponding dependence plots (mainframe of Fig. 1C and D) yields the $\Delta V_{h}\left(\equiv \partial v/\partial c_{bh}\right)$, and the changes in molar adiabatic compressibility, $\Delta K_{Sh}$ $\left(\equiv \partial k_{S}/\partial c_{bh}\right)$. It was considered that both $\Delta V_{h}$ and $\Delta K_{Sh}$ follow the similar functional relationships with $c_{bh}\left(t\right)$ as in Eq. (2), the progress of hydrolysis can also be recalculated iteratively from measured real-time profiles of volume and compressibility using:

(16) $$c_{bh}\left(t_{n+1}\right)=c_{bh}\left(t_{n}\right)\frac{v\left(t_{n+1}\right)-v\left(t_{n}\right)}{\Delta V_{h}\left(t_{n}\right)};\;c_{bh}\left(t_{n+1}\right)=c_{bh}\left(t_{n}\right)\frac{k_{S}\left(t_{n+1}\right)-k_{S}\left(t_{n}\right)}{\Delta K_{Sh}\left(t_{n}\right)}$$

where $v_{0}$ and $k_{S0}$ are the specific volume and adiabatic compressibility of the medium, respectively. Similarly, the evolving values of $\Delta V_{h}$ and $\Delta K_{Sh}$ can be determined throughout the hydrolysis process. The dependence of $\Delta V_{h}$ and $\Delta K_{Sh}$ on $c_{bh}\left(t\right)$ is also not strictly linear over the full hydrolysis range, particularly in phosphate buffer above pH 7, where the ionization correction becomes significant (Dizon & Buckin, 2023).

Accordingly, $\Delta V_{h}$ and $\Delta K_{Sh}$ can be expressed as the sum of intrinsic and ionization components following the formalism of Eq. (4) (Materials and Methods, section 2.4), with the parameter $\Delta a_{h}$ replaced by either $\Delta V_{h}$ or $\Delta K_{Sh}$. The ionization terms $\Delta V_{I}$ or $\Delta K_{SI}$ were then evaluated similarly from the pH-dependent volumetric and compressibility properties of the buffer species and the titratable amino acid side chains involved in ionization reaction during hydrolysis, using the published ionization parameters (Table S1 (Dizon & Buckin, 2023)) derived from partial molar volume and compressibility data for model amino acids and buffer components. The ionization contributions were calculated assuming additive, independent group contributions transferable from model compounds, which is valid insofar as the side-chain environments in the hydrolysate resemble those of free amino acids in dilute solution. Similarly, the intrinsic contributions $\Delta V_{P}$ and $\Delta K_{\mathrm{SP}}$ were extracted by fitting the measured time-dependent changes in $\delta v\left(t\right)$ and $\delta k_{S}\left(t\right)$ to the ultrasonically derived values of $c_{\mathrm{bh}}\left(t\right)$ using the iterative numerical procedure involving Eq. (16). The fitted results are shown in Table 1. Recalculation into the corresponding $\Delta a_{P}$ ($=\Delta V_{P}/v_{0}-\Delta K_{SP}/\left(2k_{S0}\right)$, Eq. S3) yields values of 0.087 ± 0.004 kg mol^−1^, 0.085 ± 0.004 kg mol^−1^, and 0.078 ± 0.003 kg mol^−1^ for phosphate pH 7.8, Tris pH 7.8, and phosphate pH 7.0, respectively, in close agreement with the previously published $\Delta a_{P}$ values (Dizon & Buckin, 2023).

**Table 1:** Change in apparent molar volume, $\Delta V_{P}$, apparent molar adiabatic compressibility, $\Delta K_{S\;P}$, and apparent molar adiabatic relaxation compressibility $\Delta K_{S\mathrm{rel}}$ during the α–chymotrypsin hydrolysis of β–lactoglobulin in 0.1 M phosphate pH 7.8 and pH 7.0, and 0.1 M Tris pH 7.8 and at 25 °C.

	$\Delta V_{P}$ (x10^−6^),m^3^ mol^−1^	$\Delta K_{S\;P}$(x10^−14^),m^3^ mol^−1^ Pa^−1^	$\Delta a_{P}$,kg mol^−1^	$\Delta K_{S\mathrm{rel}}$(x10^−14^),m^3^ mol^−1^ Pa^−1^
Phosphate, pH 7.8	−24.5 ± 2	−9.9 ± 0.26	0.087 ± 0.004	2.55 ± 0.1
Tris, pH 7.8	−23.1 ± 2	−9.6 ± 0.26	0.085 ± 0.004	-⁎
Phosphate, pH 7	−27.0 ± 1	−9.3 ± 0.26	0.078 ± 0.003	3.39 ± 0.1

⁎ negligibly small.

The negative values of $\Delta V_{P}$ and $\Delta K_{\mathrm{SP}}$ are consistent with the expected net decreases in volume and compressibility accompanying peptide bond hydrolysis. The changes arise from incorporation of one water molecule from the bulk into the cleaved peptide bond, consuming it from the bulk water phase, and electrostriction of water molecules in the hydration shells of the newly formed C-terminal $P_{1}-COO^{-}$, and N-terminal, $P_{2}-{{\mathrm{NH}}_{3}}^{+}$ groups. Significant differences in both $\Delta V_{P}$ and $\Delta K_{\mathrm{SP}}$, and thus $\Delta a_{P}$, were observed between phosphate buffers at pH 7.8 and pH 7.0 (duplicate measurements, p < 0.05). In contrast, no significant differences in either parameter were detected between phosphate and Tris buffers at pH 7.8 (duplicate measurements, p > 0.05), indicating that pH had a greater influence on the volumetric and compressibility changes during hydrolysis than buffer type. This likely reflects differences in intrinsic structural and hydration properties between the dominant monomeric state of β–lactoglobulin at pH 7.8 and dimeric state at pH 7.0. In contrast, the negligible difference between phosphate and Tris buffers at pH 7.8 indicates that, at the same pH and oligomeric state, the intrinsic volume and compressibility effects of peptide bond cleavage are independent of buffer type.

**Fig. 2:**
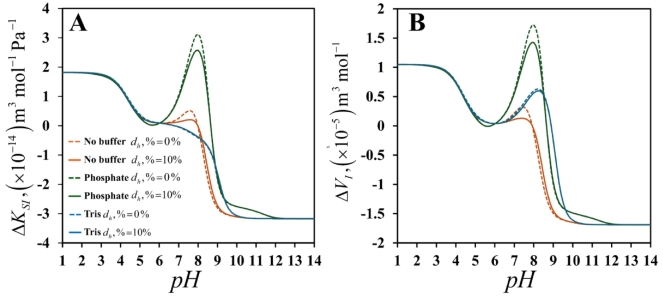
Dependence of ionic contribution $\Delta V_{I}$ and $\Delta K_{\mathrm{SI}}$ to volume and compressibility, respectively on pH at two degrees of hydrolysis $d_{h},\%$**=** 0% and 10% of β–lactoglobulin at 25 °C in unbuffered medium, 0.1 M phosphate and 0.1 M Tris buffers. The concentration of protein is 1% w/w.

To assess how the ionization contribution varies with pH and buffer composition, both $\Delta V_{I}$ and $\Delta K_{SI}$ were simulated across the whole pH range, for both unhydrolyzed and partially hydrolyzed β–lactoglobulin at degree of hydrolysis $d_{h},\%$ = 10% (Eq. (6)) in phosphate and Tris buffer, and unbuffered media as shown in Fig. 2. In the pH range of 6.5–8.5, both parameters attain their maxima near pH **≈** 8.0 in phosphate buffer. The maximum $\Delta K_{SI}$ peak observed in phosphate buffer is absent in Tris, although the corresponding $\Delta V_{I}$ maximum is shifted to pH **≈** 8.0. A progressive shift in both parameters with increasing degree of hydrolysis in phosphate buffer and, to a lesser extent, in unbuffered media, while no significant effect is observed in Tris. These trends confirm that changes in pH and the accumulation of protonated amino groups ($P_{2}-{NH_{3}}^{+}$) markedly shift $\Delta V_{I}$ and $\Delta K_{SI}$ in phosphate-buffered media within the investigated pH window. Notably, this pH range spans both the optimal activity region of α–chymotrypsin and the relevant $pK_{A}$ values of the newly formed N-terminal amino groups, as well as those of the phosphate buffer system.

**Fig. 3:**
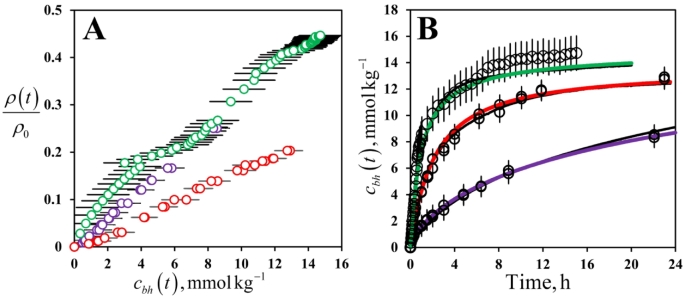
(A) Relationship between the relative density change $\delta \rho \left(t\right)/\rho_{0}$ and $c_{bh}\left(t\right)$ determined experimentally using the TNBS method for hydrolysis in phosphate buffer at pH 7.8 (red circles) and pH 7.0 (purple circles), and the continuous pH monitoring method for hydrolysis in Tris buffer at pH 7.8 (green circles). (B) Comparison between the hydrolysis profiles recalculated from ultrasonic velocity and density measurements and the experimentally determined concentrations of hydrolyzed peptide bonds. The coloured continuous lines represent the profiles recalculated from density measurements for hydrolysis in phosphate buffer at pH 7.8 (red) and pH 7.0 (purple), and in Tris buffer at pH 7.8 (green). The continuous black lines represent the profiles recalculated from ultrasonic velocity measurements at 15.5 MHz using the HR-US system, while the black circles represent the experimental calibration data obtained using the TNBS and continuous pH monitoring methods. Error bars represent propagated standard errors derived from duplicate measurements and baseline correction, shown as 95% confidence intervals. (For interpretation of the references to colour in this figure legend, the reader is referred to the web version of this article.)

To demonstrate the applicability of high-precision densitometry as an independent real-time analytical tool for monitoring enzymatic hydrolysis, similar iterative procedure of Eqs. (16) was applied to recalculate $c_{\mathrm{bh}}\left(t\right)$ directly from the measured density profiles. The measured density profiles were first calibrated by $c_{bh}\left(t\right)$ determined independently by the TNBS colorimetric method for phosphate buffer (pH 7.0 and pH 7.8) and by continuous pH monitoring for Tris buffer (pH 7.8) (Fig. S2). This establishes the relationship between $\delta \rho \left(t\right)/\rho_{0}$ and $c_{\mathrm{bh}}\left(t\right)$ shown in Fig. 3A. The slope of the plot yields the density concentration increment $\Delta {\left[\rho \right]}_{h}$ ($\equiv \partial \rho /\partial c_{\mathrm{bh}}$), whose reciprocal corresponds to $\Delta V_{h}$, which was subsequently used to recalculate real-time $c_{\mathrm{bh}}\left(t\right)$ profiles (Fig. 3B) from the density measurement via Eq. (16). The close agreement between the densitometrically recalculated and independently determined hydrolysis profiles supports the validity of the volumetric approach. The recalculation requires only the volumetric ionization parameters of the amino acid functional groups and buffer species (Table S1) and the intrinsic structural contribution, which are widely available in the literature or can be derived from the reported datasets (Dizon & Buckin, 2023).

The present validation of the calculation algorithm was carried out for the buffer compositions and protein concentration investigated here, but it is expected to be transferable to other proteolytic systems of comparable cleavage site specificity, provided that aggregation does not substantially contribute to the measured volumetric and compressibility changes and that the relevant thermodynamic parameters are independently determined using calibration methods such as TNBS assay or continuous pH monitoring. The accuracy of the ionization corrections depends on the availability of reliable partial molar volume and compressibility data for all titratable species present. Consequently, titratable groups lacking literature parameters, or systems exhibiting significant deviations from ideal-solution behavior, would introduce corresponding systematic uncertainties into the calculated proportionality coefficients.

### 3.2 Ultrasonic relaxation profiles

The multi-frequency measurement capability of HR-US enables the ultrasonic relaxation analysis of protein hydrolysis, providing insights into fast proton-transfer processes accompanying enzymatic peptide bond hydrolysis. In the present study, the relaxation behavior was analyzed using a dominant single-relaxation approximation corresponding to proton transfer between phosphate buffer species and the newly formed $P_{2}-{NH_{3}}^{+}$groups (Reaction A). Although analogous proton-transfer equilibria may occur in Tris buffer, their contribution to the measured ultrasonic relaxation is negligible under the present experimental conditions (Dizon & Buckin, 2025). This difference arises because the phosphate equilibrium possesses a substantially larger reaction capacity and associated adiabatic volume change, resulting in a significantly greater relaxation amplitude. Previous analysis of whey-protein hydrolysis in 0.1 M phosphate buffer demonstrated that the reaction capacities of competing side-chain proton-exchange equilibria (Reaction B) are negligible relative to that of Reaction A over the entire hydrolysis range investigated (Dizon & Buckin, 2025). Under these conditions, Reaction A dominates the relaxation process and therefore determines the frequency dependence of the measured ultrasonic parameters.

A similar quantitative ultrasonic relaxation analysis (Section D in SM) was carried out for the hydrolysis of β–lactoglobulin in phosphate buffer pH 7.0 using ultrasonic data acquired at 2.9 MHz, 4.8 MHz, 7.9 MHz and 15.5 MHz (Fig. S1). At this pH, the protonation states of the phosphate buffer and the newly formed $P_{2}-{NH_{3}}^{+}$ groups simultaneously maximize the reaction capacity term $\Gamma_{S}$, which governs the magnitude of the observable relaxation contribution (Dizon & Buckin, 2025), while the strong phosphate buffering capacity minimizes pH drift during hydrolysis. Changes in reactant and product concentrations during hydrolysis shifts $\Gamma_{S}$, producing corresponding shifts in the ultrasonic relaxation frequency $f_{rel}$. The parameter $f_{\mathrm{rel}}$ was determined from the slope of the linear relationship between Δu and $\Delta \alpha /f^{2}$ (Eq. S5) at three selected reaction times, 50th min ($c_{\mathrm{bh}}$ = 1.0 mmol kg^−1^), 300th mins ($c_{bh}$ = 4.0 mmol kg^−1^) and 1300th mins ($c_{bh}$ = 8.7 mmol kg^−1^) (Fig. S3B, linear regressions yielded $R^{2}$ values ranging from 0.89 to 0.91). The calculated $f_{rel}$ values ranged from 2.4 to 2.8 MHz, corresponding to a relaxation time $\tau \left(=1/2\pi f_{rel}\right)$ ≈ 6 ± 1 × 10^−8^ s. These values are notably higher than the range of relaxation frequency observed at pH 7.8 for the same proteins (Dizon & Buckin, 2025), consistent with high $\Gamma_{S}$ at pH 7.0. Furthermore, the measured ultrasonic velocity at 15.5 MHz differed by less than 0.8% from the high-frequency limit (Fig. S3B), indicating that relaxation contributions at this frequency are negligible and thereby validating its use as the calibration frequency in Section 3.1.

The linear plot of $f_{rel}$ against the concentration factor $\left(c_{H_{2}PO_{4}^{-}T}\left(x_{H_{2}PO_{4}^{-}}/x_{NH_{3}^{+}}\right)+c_{NH_{3}^{+}T}\left(1-x_{NH_{3}^{+}}/1-x_{H_{2}PO_{4}^{-}}\right)\right)$ (Fig. S3C, $R^{2}$ ≥ 0.99), yields the slope corresponds to the effective forward reaction rate constant $k_{f}^{0}\gamma_{H_{2}PO_{4}^{-}}\gamma_{NH_{2}}/\gamma_{\left\{T\right\}}$ = 29 ± 3 × 10^7^ L mol^−1^ s^−1^ (Eq. S6). Using an activity coefficient correction term $\gamma_{H_{2}{\mathrm{PO}}_{4}^{-}}\gamma_{{\mathrm{NH}}_{2}}/\gamma_{\left\{T\right\}}$ for the non-ideal solution behavior via activity coefficients with an estimate of 3, the forward rate constant $k_{f}^{0}$ ∼ 9.7 ± 1.2 × 10^7^ L mol^−1^ s^−1^, in close agreement with 9.4 × 10^7^ L mol^−1^ s^−1^ reported at pH 7.8 (Dizon & Buckin, 2025). As these values represent effective rate constants averaged over a heterogeneous hydrolysate population, they should not be interpreted as elementary microscopic rate constants. The close agreement between pH 7.0 and pH 7.8 (duplicate measurements, p > 0.05) demonstrates that $k_{f}^{0}$ is effectively pH-independent within the phosphate buffering region and supports the applicability of the ultrasonic relaxation approach throughout the activity range of α–chymotrypsin.

**Fig. 4:**
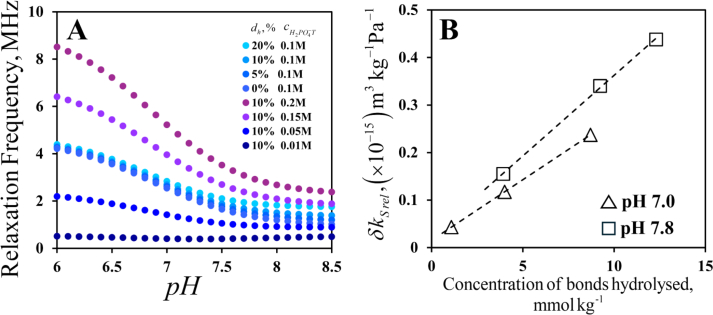
(A) Simulated pH profile of $f_{\mathrm{rel}}$ for β–lactoglobulin, illustrating the effects of degree of hydrolysis ($d_{h},\%$) and phosphate concentration. (B) Concentration profile of the change in specific adiabatic relaxation compressibility during hydrolysis at pH 7.0 and 7.8. The slope of the linear plot corresponds to the change in apparent molar adiabatic relaxation compressibility of hydrolysis $\Delta K_{S\mathrm{rel}}$. Error bars (95% confidence intervals) are smaller than the symbol size and are therefore not visible.

Using the estimated $k_{f}^{0}$ and the concentration increment of ionization parameters (Table S1), the pH profile of $f_{rel}$ was simulated across the phosphate buffering region and enzyme's activity window, at selected $d_{h},\%$ (proportional to $c_{NH_{3}^{+}T}$) and phosphate concentrations, $c_{H_{2}PO_{4}^{-}T}$ (Fig. 4A). The resulting profiles exhibit a characteristic sigmoidal dependence, with $f_{\mathrm{rel}}$ reaching its maximum near pH 7, where $\Gamma_{S}$ is highest. Increasing the total phosphate concentration at a constant $d_{h},\%$ markedly increases $f_{rel}$ and amplifies the difference between pH 6 and pH 8.5, whereas changes in $d_{h},\%$ exert a comparatively minor effect because the concentration of $P_{2}-{NH_{3}}^{+}$ groups remains substantially lower than that of the buffer throughout the reaction. These simulations highlight the dominant influence of buffer concentration and protonation equilibria on ultrasonic relaxation behavior and provide a quantitative framework for predicting relaxation properties across phosphate-buffered systems within the investigated pH 6–8.5 and protein concentration ranges, without dedicated relaxation measurements.

The magnitude of the relaxation contribution to the ultrasonic parameters were assessed through the relaxation compressibility coefficient: $k_{S\;rel}=\Gamma_{S}{\left(\Delta V_{S}\right)}^{2}\left(1/RT\right)$, where $\Delta V_{S}$ is the adiabatic volume change associated with the proton transfer reactions. This adiabatic volume change arises from pressure-induced oscillation of the reaction equilibrium position during the propagation of the ultrasonic wave. Simultaneous fitting of the multi-frequency ultrasonic attenuation profiles at 2.9, 4.8, 7.9, and 15.5 MHz to the single-relaxation model (Eq. S7, Fig. S3D) using a nonlinear least-squares procedure ($R^{2}$ > 0.92), incorporating the concentration-dependent reaction capacity terms and the ionization equilibria of phosphate species and hydrolysate amino groups, yielded $\Delta V_{S}$ value equal to 20.5 ± 2 × 10^−6^ m^3^ mol^−1^. The fitted value is also in close agreement with the previously reported average of $\left|\Delta V_{S}\right|$ = 21 ± 3 × 10^−6^ m^3^ mol^−1^ for whey proteins at pH 7.8, and in agreement with thermodynamic volume data for the proton transfer between $H_{2}PO_{4}^{-}$ and N-terminal amino groups (Dizon & Buckin, 2025). The small difference between the measured value and the ionic-strength-corrected literature value is accounted for by the ionic contribution $\Delta V_{\mathrm{SI}}$= 2 × 10^−6^ m^3^ mol^−1^ at I = 0.282 mol L^−1^. The invariance of $\Delta V_{S}$ with pH confirms that the underlying proton-transfer step retains essentially the same thermodynamic character throughout the phosphate buffering region.

The slope of linear dependence between the $\delta k_{S\mathrm{rel}}$ and $c_{\mathrm{bh}}\left(t\right)$ (Fig. 4, $R^{2}$ ≥ 0.99) yields the change in apparent molar adiabatic relaxation compressibility $\Delta K_{S\mathrm{rel}}\;\left(\equiv \partial k_{S\mathrm{rel}}/\partial c_{\mathrm{bh}}\right)$ equal to 2.55 ± 0.1 × 10^−14^ m^3^ mol^−1^ Pa^−1^ and 3.39 ± 0.1 × 10^−14^ m^3^ mol^−1^ Pa^−1^ for pH 7.8 and 7.0, respectively. These values have not been reported previously for any protein hydrolysis system and constitute a new thermodynamic characterization of thepH-dependent ultrasonic response. As $\Delta K_{S\mathrm{rel}}$ is determined by the second derivative of thermodynamic potentials, i.e., ${\left(\partial^{2}G/\partial P\partial \zeta \right)}_{S}$ and ${\left(\partial^{2}G/\partial \zeta^{2}\right)}_{S}$, where G is the Gibb's free energy and ζ is the reaction extent, it is extremely sensitive to the chemical equilibrium shifts of the reactant and product components. In this context, $\Delta K_{S\mathrm{rel}}$ is proportional to $\Gamma_{S}{\left({\mathrm{\Delta V}}_{S}\right)}^{2}$, and is therefore generally positive, with its magnitude directly controlled by the reaction capacity at each pH. Hence, the higher value of $\Delta K_{S\mathrm{rel}}$ at pH 7.0 compared to pH 7.8 directly reflects the greater reaction capacity $\Gamma_{S}$ at pH 7.0, where the protonation states of both phosphate and N-terminal groups are optimally balanced for fast proton exchange. More generally, $\Delta K_{S\mathrm{rel}}$ characterizes the coupling between pressure perturbations and chemical equilibrium and may therefore provide a useful parameter for investigating protein hydration, intermolecular interactions, and related dynamic processes under non-equilibrium or pressure-dependent conditions.

### 3.3 Structural profiles

#### 3.3.1 Correlation of hydrolysis kinetics with secondary structural changes

Following the real-time quantification of peptide bond hydrolysis by HR-US, Far-UV Circular Dichroism (CD) spectroscopy measurement was employed to monitor secondary structural transitions during the α–chymotrypsin hydrolysis of β–lactoglobulin and to establish how peptide bond cleavage within the primary sequence drives the secondary structural transitions (Güler et al., 2011). It should be noted that the volumetric and compressibility contributions arising from secondary structural rearrangements are considerably smaller than the hydration effects associated with peptide bond cleavage (Mori et al., 2006), thus supporting the use of precision ultrasonic measurements of hydration changes as a quantitative measure of the degree of hydrolysis, while the CD method offers an independent structural measurement linking structural unfolding with the degree of hydrolysis.

**Fig. 5:**
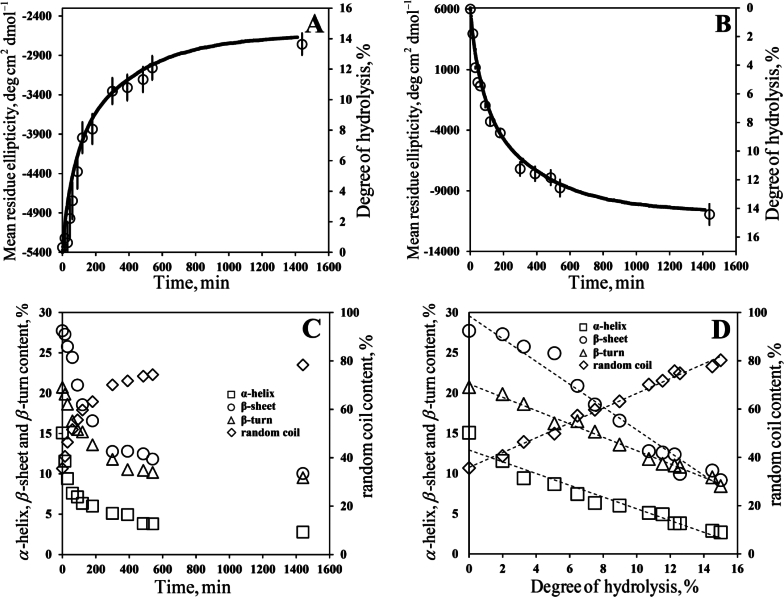
Comparison of time profiles of the changes in mean residual ellipticity at 195 nm (A, primary y-axis) and 218 nm (B, primary y-axis) measured by CD spectroscopy and ultrasonically measured degree of hydrolysis (secondary y-axis of Panel A and B) of β–lactoglobulin (1% *w*/w) catalyzed by α–chymotrypsin (0.01% w/w), in 0.1 M phosphate buffer pH 7.8. Error bars represent 95% confidence intervals. Panels C and D show the evolution of α–helix, β–sheet, β–turn and random coil contents as a function of hydrolysis time and degree of hydrolysis, respectively. Error bars represent 95% confidence intervals and are smaller than the symbol size.

The native β–lactoglobulin spectrum (Fig. S4) exhibited a positive maximum at ∼195 nm (α–helix), and a characteristic negative band around 218 nm (antiparallel β–sheet) (Ma et al., 2018). Secondary structure deconvolution using CDPro (Sreerama, 2025), applying the SELCON3, CONTINLL, and CDSSTR algorithms simultaneously against the SP175 reference database (47 proteins, 190–240 nm range), yielded a native β–lactoglobulin composition of 15.1 ± 0.4% α–helix, 27.7 ± 0.9% β–sheet, 20.7 ± 1.1% β–turn, and 35.6 ± 2.2% random coil. These values are consistent with literature ranges of 16–18% α–helix and 33–35% β–sheet content, and β–lactoglobulin's total β-structure content (β–sheet + β–turn ≈ 48%) falls within the expected range of 40–60% (Rahmani-Manglano et al., 2022; Zhai et al., 2011). Variations in reported secondary structure contents are expected owing to differences in experimental conditions, reference datasets, and deconvolution algorithms. Fig. 5A and B illustrate the time-dependent ellipticity changes at 195 nm and 218 nm, respectively, compared with the ultrasonically measured $d_{h}$. After addition of α–chymotrypsin, the decrease in the amplitude of 190–195 nm band indicated the reduction in α–helix content, while the blue-shift of the 210–220 nm band toward ∼200 nm indicated the β–sheet disruption and the accumulation of random coils (Rahmani-Manglano et al., 2022). The corresponding evolution of secondary structural elements over the hydrolysis reaction is presented in Fig. 5C, while their dependence on the degree of hydrolysis $d_{h}$ in Fig. 5D.

All ordered motifs (α–helix, β–sheet, β–turn) decreased continuously, accompanied by a corresponding increase in random coil content during hydrolysis (Güler et al., 2016; Zhang et al., 2024). A notable kinetic feature is that the reduction in α–helical content precedes the disruption of β–sheet structures across the duplicates. For $d_{h},\%$ > 2%, both α–helix and β–sheet decrease linearly ($R^{2}$ = 0.97–0.98) with $d_{h}$. This observation suggests that the initial cut by α–chymotrypsin in the surface of the protein simultaneously disrupts the α–helix which then exposes the hydrophobic core, facilitating the subsequent disruption of the rigid β–sheet structure. In parallel, the linear increase in random coil content with degree of hydrolysis ($R^{2}$ = 0.99) confirms that hydrolysis drives a steady, irreversible transition from the ordered globular architecture to disordered peptide fragments.

Although CD spectroscopy has previously been used to investigate structural changes in β–lactoglobulin hydrolysis, the present study correlates continuously monitored secondary structural changes with the real-time degree of hydrolysis measured independently by HR-US. This approach enables structural transitions to be analyzed as a function of degree of hydrolysis rather than reaction time alone. Previous studies reporting sequential α–helix and β–sheet disruption in protein hydrolysis relied on time-course profiles only, where the degree of hydrolysis was either not quantified in parallel or was measured separately using discontinuous assays such as TNBS or OPA (Güler et al., 2016; Rahmani-Manglano et al., 2022). By combining continuous hydrolysis quantification with simultaneous structural measurements, the present methodology provides a direct kinetic–structural framework for relating peptide bond cleavage to changes in protein secondary structure. Overall, the results reveal a gradual and predictable transition from the native globular state toward a disordered peptide population as hydrolysis progresses.

#### 3.3.2 Intrinsic tryptophan fluorescence profiles

Tertiary structural transitions accompanying hydrolysis were monitored by intrinsic fluorescence spectroscopy. As hydrolysis progressed, a bathochromic shift (red shift) in the emission maximum $\lambda_{\max}$ was observed (Fig. S5), reflecting the transition of tryptophan (Trp) residues from the buried, non-polar hydrophobic core to a solvent-exposed, polar environment (Vorob'ev et al., 2011). The spectral shift is accompanied by spectral broadening and intensity increase, indicating disruption of native quenching interactions and increasing heterogeneity in tryptophan microenvironments during hydrolysis. The observed spectra therefore reflect the combined fluorescence contributions of native β–lactoglobulin and its hydrolysis products (Lyu et al., 2023).

The measured intrinsic tryptophan fluorescence spectra were further analyzed using the two-step “demasking – hydrolysis” proteolysis model proposed by Vorob'ev et al. (Vorob'ev, 2020; Vorob'ev, 2022; Vorob'ev et al., 2011). For β–lactoglobulin, the following calculation algorithm is briefly outlined in Section 2.8, and this framework treats the observed fluorescence spectrum as arising from a mixture of native (Trp-masked) and demasked (Trp-exposed) states and assumes that the dominant fluorescence emission can be described by the two-step proteolysis model. Intermediate conformations with distinct tryptophan environments are assumed not to accumulate to concentrations sufficient to contribute substantially to the measured emission maximum. Under these assumptions, the limiting emission maximum $\lambda_{\max}$ was estimated by extrapolation of the hydrolysis profile to infinite reaction time and represents the limiting fully demasked state. This approximation is particularly reasonable for β–lactoglobulin, which contains two Trp residues (Trp-19 and Trp-61) buried within the β–barrel hydrophobic core (Kontopidis et al., 2004; Vorob'ev, 2022). Hence, this is not applicable to proteins lacking Trp residues or in systems where intermolecular fluorescence energy transfer between liberated peptide fragments becomes significant. Furthermore, the absence of late-stage fluorescence quenching suggests that proteolysis proceeds without substantial peptide reassociation or reburial of newly exposed Trp residues.

**Fig. 6:**
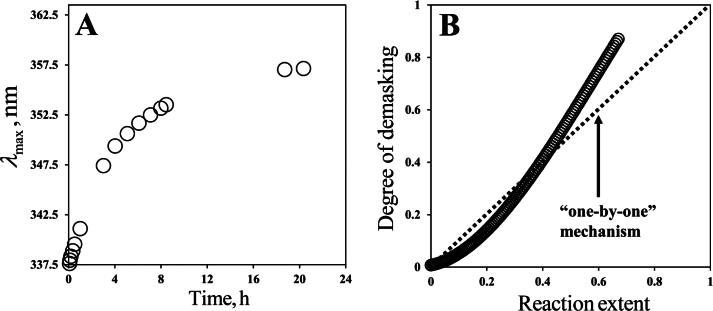
(A) Time evolution of the fluorescence emission peak maximum $\lambda_{\max}$ (primary y-axis). (B) Correlation between the experimentally determined degree of demasking ($1-x\left(t\right)=\left(\lambda_{\max}\left(t\right)-\lambda^{0}\right)/\left(\lambda^{\infty }-\lambda^{0}\right)$) and the reaction extent (degree of hydrolysis) of α–chymotrypsin hydrolysis of β–lactoglobulin, based on the theoretical specific cleavage sites (Phe–X, Trp–X, Tyr–X, Met–X, and Leu–X). The dashed lines represent a ‘one-by-one’ proteolytic mechanism. Hydrolysis condition: 1% w/w β–lactoglobulin treated with 0.01% w/w α–chymotrypsin in 0.1 M phosphate buffer pH 7.8. 25 °C.

The initial emission maximum wavelength $\lambda_{\max}^{0}$ value of 337.24 ± 0.5 nm is a characteristic of tryptophan residues buried within the hydrophobic calyx of native β–lactoglobulin (Vorob'ev, 2022). During hydrolysis, a progressive red-shift in $\lambda_{\max}$ was observed (Fig. 6A), indicating increased solvent exposure of tryptophan residues associated with structural unfolding and peptide demasking. The corresponding degree of demasking $1-x\left(t\right)$ was calculated using Eq. (13) using $\lambda_{\max}^{0}$ = 337.2 ± 0.5 nm and $\lambda_{\max}^{\infty }$ = 361.7 ± 0.8 nm (determined by extrapolation to infinite hydrolysis time). Fig. 6B correlates the demasking profile with the reaction extent (ξ), defined as the fraction of susceptible cleavage sites hydrolyzed: $\xi =d_{h}/0.2236$, where $d_{h}$ was determined directly from the ultrasonically measured concentration of cleaved peptide bonds. The factor 0.2236 corresponds to the fraction of peptide bonds susceptible to α–chymotrypsin cleavage in β–lactoglobulin, based on 36 potential cleavage sites among 161 peptide bonds assuming specificity toward Phe, Trp, Tyr, Met, and Leu residues (Vreeke et al., 2023). Propagation of these uncertainties yields an approximate ± 3% relative uncertainty in ξ at intermediate demasking levels, under the assumption that all susceptible sites are accessible during hydrolysis.

The complementary fluorescence measurement with HRUS revealed the non-linear relationship between the reaction extent and the degree of demasking (Fig. 6B), which is partially attributed to “one-by-one” mechanism according to Linderstrøm-Lang theory (Vorob'ev, 2022, 2025). According to “one-by-one” mechanism, the rate of demasking is directly associated with the rate of peptide bond cleavage without the accumulation of partially demasked intermediates. Combining the HR-US real-time degree of hydrolysis profile with simultaneously acquired intrinsic tryptophan fluorescence data provides a higher-resolution experimental test of this mechanistic framework than previously possible and enables quantitative characterization of the relationship between peptide bond cleavage and tertiary structural disruption throughout the hydrolysis process.

### 3.4 Hydrodynamic profiles

#### 3.4.1 Light scattering profiles

**Fig. 7:**
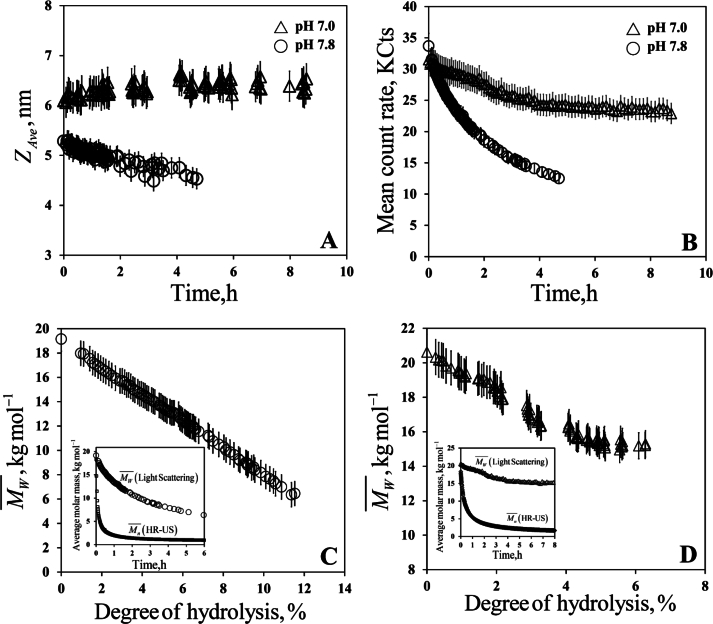
Light scattering analysis of α–chymotrypsin hydrolysis of β–lactoglobulin in 0.1 M phosphate buffer, 25 °C: (A) and (B) illustrate the time evolution of measured $Z_{Ave}$ and measured light scattering intensity data (expressed as mean count rate), respectively, at pH 7.8 (triangle) and pH 7.0 (circle), and (C) and (D) display the dependence plot (main frame) of calculated apparent $\bar{M_{W}}$ from light scattering intensity data with ultrasonically measured concentration of peptide bonds hydrolyzed in β–lactoglobulin at pH 7.8 and pH 7.0, respectively. Insets (C) and (D) show the original time profiles of apparent $\bar{M_{W}}$ measured by light scattering technique and $\bar{M_{n}}$ measured by HR-US technique. Error bars represent the error associated with the 95% confidence interval.

Real-time single-angle static and dynamic light scattering measurements were performed to monitor changes in apparent molecular size and hydrodynamic dimensions accompanying enzymatic hydrolysis. Fig. 7A and B illustrate the evolution of the intensity-weighted mean hydrodynamic size $Z_{\mathrm{Ave}}$ and scattered-light intensity $I_{P}$ (expressed photon count rates), respectively, during α–chymotrypsin hydrolysis of β–lactoglobulin at pH 7 and pH 7.8. Initial $Z_{ave}$ values of 5.3 ± 0.2 nm at pH 7.8 and 6.1 ± 0.3 nm at pH 7.0 were consistent with predominantly monomeric and dimeric populations states of native β–lactoglobulin, respectively (Elofsson et al., 1996). The measured scattering intensity was further used to derive the apparent weight-average molar mass $\bar{M_{W}}$ of unhydrolyzed β–lactoglobulin and the subsequent hydrolysate mixtures using the Rayleigh scattering approximation (Eq. (14)), with toluene as the calibration reference $I_{\text{toluene}}$ (Eq. S10). The initial apparent $\bar{M_{W}}$ values of 18.96 kg mol^−1^ at pH 7.8 and 23.15 kg mol^−1^ at pH 7.0 for unhydrolyzed β–lactoglobulin also confirms the presence of a minor population of dimers prior to enzyme addition.

During hydrolysis, $Z_{Ave}$ exhibited only a small decrease at pH 7.8 and no statistical change at pH 7.0 (Fig. 7A), despite the significant peptide bond cleavage was quantified. This is because the intensity-weighted $Z_{Ave}$ is dominated by residual intact globular protein molecules at each time point. The relatively small change in average hydrodynamic dimensions despite extensive hydrolysis is consistent with a mechanism in which intact protein molecules coexist with much smaller peptide products, while intermediate-sized species do not accumulate to high concentrations (Vorob'ev, 2022). In contrast, both the scattered light intensity $I_{P}$ and derived apparent $\bar{M_{W}}$ decreased significantly as hydrolysis progressed (Fig. 7B–D). At pH 7.8, the apparent $\bar{M_{W}}$ decreased approximately linearly with the ultrasonically measured degree of hydrolysis, consistent with a simple enzymatic depolymerization process. At pH 7.0, a similar overall trend was observed, although the data exhibited greater scatter, particularly at $d_{h,\%}$ > 2%. This increased variability could be attributed to weak and reversible intermolecular associations that become more favorable at pH 7.0 owing to reduced electrostatic repulsion. Because light scattering is highly sensitive to even minor populations of larger species, these low-level interactions produce measurable fluctuations in the signal without fundamentally altering the overall linear trend in decrease in $\bar{M_{W}}$. Accordingly, the derived $\bar{M_{W}}$ values should therefore be interpreted as indicative apparent trends rather than precise absolute molecular weights, whose absolute values carry systematic uncertainty arising from polydispersity and potential deviations from ideal behavior at pH 7.0, where weak peptide–peptide associations are evidenced. Although this limitation is most evident at pH 7.0, the same limitation applies throughout the hydrolysis process at both pH conditions. Nevertheless, the consistent monotonic decrease in apparent $\bar{M_{W}}$ across all experiments provides robust evidence of progressive protein hydrolysis.

The light scattering derived apparent $\bar{M_{W}}$ profile was compared with the ultrasonically determined number-average molecular mass, $\bar{M_{n}}$, calculated from real-time profiles of $c_{bh}\left(t\right)$ using Eq. (7). The increasing divergence throughout the hydrolysis (Fig. 7C and D insets) reflects the progressive broadening of the molecular-mass distribution. The pronounced difference in their initial rates of decrease indicates that peptide bond cleavage proceeds non-uniformly across the protein population. The more rapid decrease of $\bar{M_{n}}$ relative to $\bar{M_{W}}$ is therefore consistent with the early generation of small peptide products while a fraction of the protein population remains at comparatively high molecular mass before undergoing further hydrolysis.

#### 3.4.2 Intrinsic viscosity profiles

**Fig. 8:**
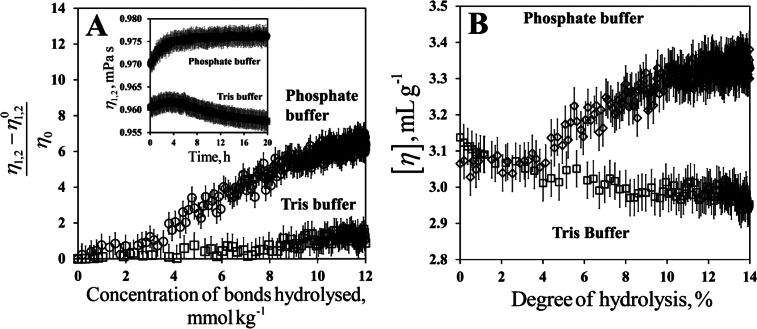
**(**A) Time profile of change in dynamic viscosity during hydrolysis of β–lactoglobulin by α–chymotrypsin in 0.1 M phosphate buffer, and in 0.1 M Tris at pH 7.8; and (B) Change in intrinsic viscosity (calculated using Solomon-Ciutǎ Equation (Eq. (15)) with the degree of hydrolysis. Error bars represent the error associated with the 95% confidence interval.

Enzymatic hydrolysis of proteins produces measurable changes in the macroscopic hydrodynamic properties of the reaction mixture. Fig. 8A illustrates the relative dynamic viscosity change $\left(\eta_{1,2}\left(t\right)-{\eta_{1,2}}^{0}\right)/\eta_{0}$, where $\eta_{1,2}\left(t\right)$ and ${\eta_{1,2}}^{0}$ are the dynamic viscosity at time t and time zero of hydrolysis, respectively, and $\eta_{0}$ is the viscosity of the corresponding buffer. Notably, a progressive increase in viscosity with increasing $c_{\mathrm{bh}}\left(t\right)$ is observed in phosphate buffer, whereas no comparable increase is detected in Tris buffer. The rise in bulk viscosity is atypical for enzymatic depolymerization processes, where viscosity generally decreases as a result of structural fragmentation and molecular size reduction (Le et al., 2025). The origin of the viscosity increase in phosphate buffer cannot be established conclusively from the present data. One plausible, though inferential, interpretation is that the viscosity rise in phosphate buffer could reflect the progressive hydrodynamic expansion of the peptide population accompanying the increasing extended random coil structure observed by CD spectroscopy and the loss of native tertiary structure indicated by intrinsic tryptophan fluorescence measurement. Such structural changes may produce conformations with larger effective hydrodynamic volumes and enhanced hydration in phosphate buffered media. By analogy, a qualitatively similar monotonic viscosity increase has been reported during the enzymatic digestion of DNA, where the transition from compact coils to extended chain conformations produces a monotonic viscosity increase due to the significant increase of the radius of gyration (Michieletto et al., 2022). However, direct comparison should be treated cautiously because proteins and nucleic acids differ fundamentally in chain architecture, charge distribution, and intermolecular interactions.

Interpreting viscosity changes in protein hydrolysates is inherently complex because it reflects coupled effects of conformational flexibility, molecular polydispersity, hydration, and intermolecular interactions (Raut & Kalonia, 2016; Zidar et al., 2020). The buffer dependence observed here suggests that phosphate ions may influence viscosity through effects on hydration and weak peptide–buffer interactions, though the presented viscosity method data do not allow these possibilities to be distinguished unambiguously.

To quantify the evolving dilute solution behavior, the intrinsic viscosity $\left[\eta \right]$ of the hydrolysis mixture from the measured $\eta_{1,2}$ was estimated from the measured dynamic viscosity using Solomon-Ciutǎ approximation (Eq. (15)) together with the corresponding ultrasonically determined $c_{\mathrm{bh}}$. The dependence of $\left[\eta \right]$ with the degree of hydrolysis ($d_{h},\%$) (Fig. 8B) in phosphate reveals a distinct three-stage non-monotonic profile - a steady regime ($d_{h},\%$ < 5%), a gradual rise (5% < $d_{h},\%$ < 14%), and a saturation plateau ($d_{h},\%$ > 14%). The boundaries between these stages are approximate, given the uncertainty scatter inherent in the single-point $\left[\eta \right]$ estimates. The observed increase in $\left[\eta \right]$ in phosphate-buffered system suggests a hydrodynamic expansion that overrides the expected effects of depolymerization alone. The saturation plateau at $d_{h},\%$ > 14% reflects that further hydrolysis cleaves predominantly already disordered peptide chains and therefore produces little additional increase in hydrodynamic volume and eventually leads to a decrease in $\left[\eta \right]$. These interpretations are inferential and would require confirmation by size-resolved techniques capable of directly monitoring the distribution of hydrolysis products. Nonetheless, the buffer-dependent viscosity response, present in phosphate but absent in Tris at the same pH, has not been previously reported for this system, and is consistent with the buffer-specific hydration and ion-interaction effects discussed above.

#### 3.4.3 Hydrodynamic parameters

The comparison of measured profiles of $d_{h}$ from HR-US and density, $Z_{\mathrm{Ave}}$ and $\bar{M_{W}}$ from light scattering method and $\left[\eta \right]$ from viscometry enables the multi-parameter analysis of hydrodynamic changes, from which hydrodynamic dimensions and volumes, as well as hydration parameters, can be derived. These calculations assume dilute-solution conditions with negligible intermolecular interactions and treat each molecular species as an effective sphere with equivalent hydrodynamic volume, together with additive molecular volumes and uniform solvent association in the hydration analysis. The ratio $\left[\eta \right]/v$ yields the estimates of universal shape factor $v_{a/b}$ (Eq. S11). For native β–lactoglobulin, $v_{a/b}$ is 3.1, consistent with a predominantly prolate ellipsoidal shape with a possible contribution from dimeric structures (Parker et al., 2005). During hydrolysis, variations in $v_{a/b}$ are determined primarily by changes in $\left[\eta \right]$, as the contribution from v is comparatively small. Accordingly, $v_{a/b}$ increases to approximately 3.4 in phosphate buffer and decreases to approximately 2.9 in Tris buffer (Fig. S6A). Within the limitations of the shape-factor model, the higher value in phosphate buffer is consistent with relatively more extended peptide conformations, whereas the lower value in Tris suggests relatively more compact fragments. This difference should be interpreted as an indication of relative trend rather than a precise geometric characterization. The product $\bar{M_{w}}\left[\eta \right]$, proportional to the effective hydrodynamic volume, decreased linearly with the degree of hydrolysis (Fig. S6B). The ratio $\bar{M_{w}}\left[\eta \right]/v_{a/b}$ estimates the average hydrated molecular volume $V_{M}$ (Eqs. S12), and the corresponding average hydrodynamic radius $R_{H}$ (Eqs. S13) of proteins. Accordingly, the $V_{M}$ and $R_{H}$ yielded a similar monotonic decrease, with $V_{M}$ and $R_{H}$ decreasing from the initial value of 31 nm^3^ to 5 nm^3^, and ∼ 2.4 nm to ∼1.3 nm, respectively.

The hydration parameter δ, estimated from partial specific volume and $\bar{M_{W}}$ (Eqs. S14) remained approximately constant throughout hydrolysis at ∼0.25 g of water per 1 g of protein with a hydration expansion factor h (Eqs. S15) in the range between 1.33 and 1.37, across all time points, despite progressive peptide bond cleavage, changing molecular size, and evolving fragment distribution. This suggests that the intrinsic water-binding capacity per unit protein mass is preserved throughout hydrolysis. To the extent that the underlying hydrodynamic assumptions remain valid, these observations are consistent with the approximation of constant hydration parameters over the hydrolysis range investigated. More generally, they suggest that the hydration shell is effectively re-established following cleavage events, resulting in a relatively constant hydration contribution on a mass basis irrespective of chain length.

Overall, because the hydrodynamic analyses were performed under dilute conditions and rely on simplifying assumptions, the absolute numerical values should be interpreted with caution. Nevertheless, the consistent monotonic trends across all hydrodynamic parameters, the clear buffer dependence, and the comparison of molecular mass averages together provide a reliable description of the depolymerization process, confirming that the protein undergoes measurable changes in molecular size and shape during enzymatic hydrolysis. The results are consistent with substantial changes in molecular size and conformation during hydrolysis, while indicating that intermolecular association, if present, remains sufficiently limited that it does not dominate the observed volumetric, ultrasonic, and hydrodynamic responses. Generally, correlating light scattering, viscometry, and ultrasonic/volumetric data against a common, independently validated extent of hydrolysis represents a multi-parameter approach to characterizing enzymatic protein hydrolysis that has not been previously demonstrated.

## 4 Conclusion

This study presents an integrated, real-time framework for monitoring the enzymatic hydrolysis of β–lactoglobulin by α–chymotrypsin, linking the progression of peptide bond hydrolysis to simultaneous changes in protein volume, compressibility, structure, and hydrodynamic behavior. High-resolution ultrasonic spectroscopy (HR-US), combined with density measurements, enabled monitoring of volume and compressibility changes and allowed quantitative separation of intrinsic contribution per bond cleavage from ionization effects. Direct validation of density-derived hydrolysis profiles against independent TNBS and continuous pH monitoring measurements confirms the validity of densitometry as a calibrated, complementary real-time monitoring tool under the stated conditions. Multi-frequency ultrasonic analysis at pH 7.0 and 7.8 yielded molar adiabatic relaxation compressibility $\Delta K_{Srel}$ values of 2–4 × 10^−14^ m^3^ mol^−1^ Pa^−1^, and confirmed pH-independence of the underlying forward rate constant $k_{f}^{0}$, extending the validated applicability of the relaxation method across the activity range of α–chymotrypsin. Complementary circular dichroism and intrinsic fluorescence measurements indicate that the progressive loss of secondary structure and increased exposure of the hydrophobic core correlates with the extent of hydrolysis. Hydrodynamic analyses reveal how these structural changes influence hydrodynamic volume and molecular dimensions. The slight increase in viscosity observed in phosphate buffer is consistent with a progressive transition from compact globular conformations toward more extended coil-like fragments, and may be further influenced by specific interactions between the hydrolysate peptides and phosphate buffer ions; however, the precise mechanistic origin of this effect cannot be established from the bulk viscometry data alone and would require confirmation by size-resolved methods.

By combining these complementary techniques and linking them to real-time information on the number of peptide bonds hydrolyzed, this work establishes a predictive framework for understanding how enzymatic hydrolysis drives the evolution of volume, structure, and hydrodynamic properties in solution. This approach provides new insight into the progression of protein hydrolysis and offers a basis for guiding the design and processing of functional protein ingredients by predicting how molecular-level changes influence solution behavior.

## Declaration of generative AI and AI-assisted technologies in the manuscript preparation process

During the preparation of this work the authors used ChatGPT and Claude in order to improve readability and language, and to perform a final proofreading of the revised manuscript and supplementary material. After using this tool, the author reviewed and edited the content as needed and takes full responsibility for the content of the publication.

## CRediT authorship contribution statement

**Mark Dizon:** Writing – review & editing, Writing – original draft, Validation, Methodology, Investigation, Formal analysis, Data curation, Conceptualization.

## Declaration of competing interest

The author declare that they have no known competing financial interests or personal relationships that could have appeared to influence the work reported in this paper.

## Data Availability

Data will be made available on request.
