# Supplementary material for: Linking enzymatic hydrolysis to structural, volumetric, and hydrodynamic evolution of β–lactoglobulin in solution
Source: Food Chem X. 2026 Jun 29;37:104152. doi: 10.1016/j.fochx.2026.104152 (PMC13356646; doi:10.1016/j.fochx.2026.104152)
Supplement: Supplementary file 1 — Supplementary material. [file mmc1.docx]

Supplementary materials

**Linking Enzymatic Hydrolysis to Structural, Volumetric, and Hydrodynamic Evolution of β-Lactoglobulin in Solution**

Mark Dizon*

*School of Chemistry, University College Dublin, Belfield, Dublin 4, Ireland*

*Department of Biomedical Science, Faculty of Health and Society, Malmö University, SE-205 06 Malmö, Sweden*

*Corresponding author: E-mail address: [mark.dizon@mau.se](mailto:mark.dizon@mau.se)

1. Table of Ionisation parameters

**Table S1** Apparent ionisation constant $pK_{A}^{app}$, change in concentration increments $\Delta a_{AH}$, molar volume $\Delta V_{AH}$ and molar adiabatic compressibility $\Delta K_{SAH}$ of ionisation for amino acid side chains and terminal groups of protein hydrolysates, phosphate, Tris, and pure water at 25 °C (Dizon & Buckin, 2023).

| Amino acid side chain or end group | Reaction of ionisation | $pK_{A}^{app}$^a^ | $\Delta a_{AH}$,  kg mol^-1 a^ | $\Delta V_{AH}$,  (x10^‒6^)  m^3^ mol^-1 a^ | $\Delta K_{SAH}$,  (x10^‒14^)  m^3^ mol^-1^ Pa^-1^ ^a^ |
| --- | --- | --- | --- | --- | --- |
| Asp | $-COO^{-}+H^{+}\to-COOH$ | 2.0 | –0.048 | 13.0 | 1.58 |
| Glu | $-COO^{-}+H^{+}\to-COOH$ | 4.2 | –0.007 | 13.0 | 1.58 |
| His | $-I+H^{+}\to-IH^{+}$ | 6.4 | 0.011 | –2.1 | –1.19 |
| Cys | $-S^{-}+H^{+}\to-SH$ | 6.9 | 0.019 | 12.4 | –0.59 |
| Tyr | $-O^{-}+H^{+}\to-OH$ | 8.0 | –0.024 | 13.5 | 3.33 |
| Lys | $-NH_{2}+H^{+}\to-NH_{3}^{+}$ | 10.3 | 0.004 | –4.0 | –2.28 |
| Arg | $=NH+H^{+}\to=NH_{2}^{+}$ | 12.0 | 0.021 | –4.0 | –2.28 |
| α–NH_2_ | $-NH_{2}+H^{+}\to-NH_{3}^{+}$ | 7.6 | 0.015 | –4.8 | –1.69 |
| α–COOH | $-COO^{-}+H^{+}\to-COOH$ | 4.45 | –0.01 | 10.5 | 1.82 |
| Phosphate 1 | $H_{2}PO_{4}^{-}+H^{+}\to H_{3}PO_{4}$ | 11.6 | –0.032 | 35.9 | 7.85 |
| Phosphate 2 | $HPO_{4}^{2-}+H^{+}\to H_{2}PO_{4}^{-}$ | 6.9 | –0.0179 | 25.6 | 3.73 |
| Phosphate 3 | $PO_{4}^{3-}+H^{+}\to HPO_{4}^{2-}$ | 2.2 | –0.0066 | 16.3 | 4.05 |
| Tris | $R-NH_{2}+H^{+}\to R-NH_{3}^{+}$ | 8.1 | 0.0129 | 4.3 | –2.31 |
| Water | $OH^{-}+H^{+}\to H_{2}O$ | 14.0 | –0.033 | –21.7 | –4.86 |

^a^Values were taken from Dizon & Buckin (Dizon & Buckin, 2023).

1. Supplementary Figures

Figure S1 Evolution of ultrasonic velocity (main frame) and attenuation (insets) during hydrolysis of β‒lactoglobulin by α‒chymotrypsin in 0.1 M phosphate buffer $pH$ 7.0 at 25 °C, measured at four selected ultrasonic frequencies – 2.9 MHz (green symbols), 4.8 MHz (sky blue symbols), 7.9 MHz (orange symbols) and 15.5 MHz (purple symbols), by HR-US.


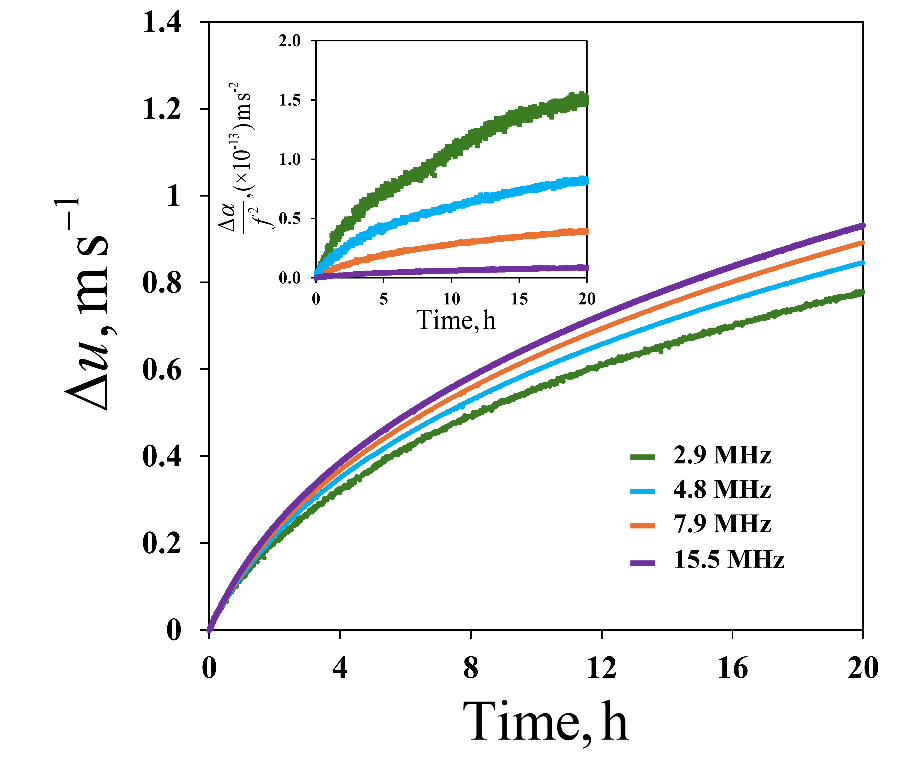


Figure S2 Compiled changes in density profiles during hydrolysis of 1% (w/w) β‒lactoglobulin by 0.01% (w/w) α-chymotrypsin, together with experimentally determined concentrations of peptide bond hydrolysed using TNBS assay in 0.1 M phosphate buffer at $pH$ 7.8 (A) and $pH$ 7.0 (B), and using continuous $pH$ monitoring in 0.1 M Tris buffer at $pH$7.8 (C), at 25 °C. Error bars indicate 95% confidence intervals.


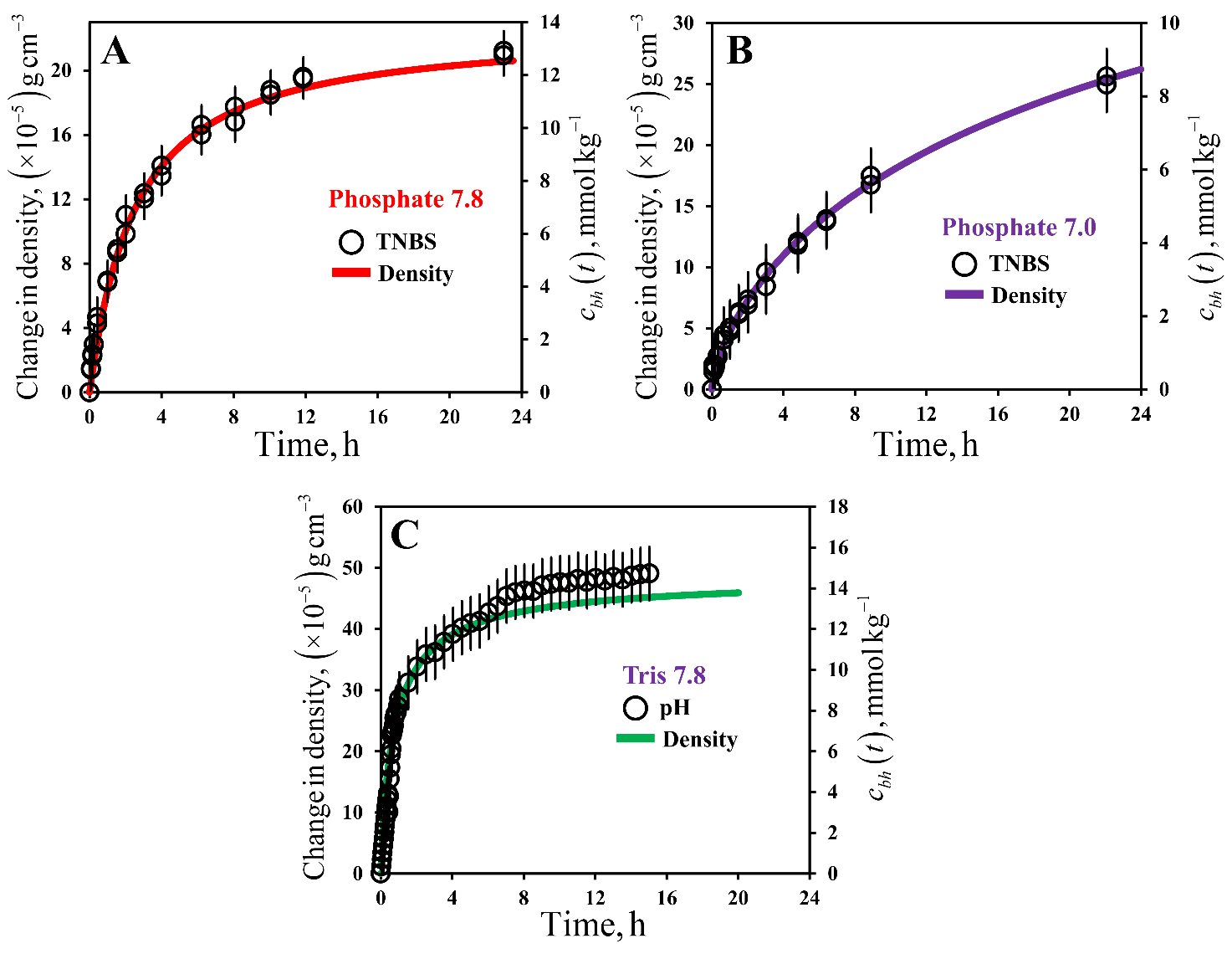

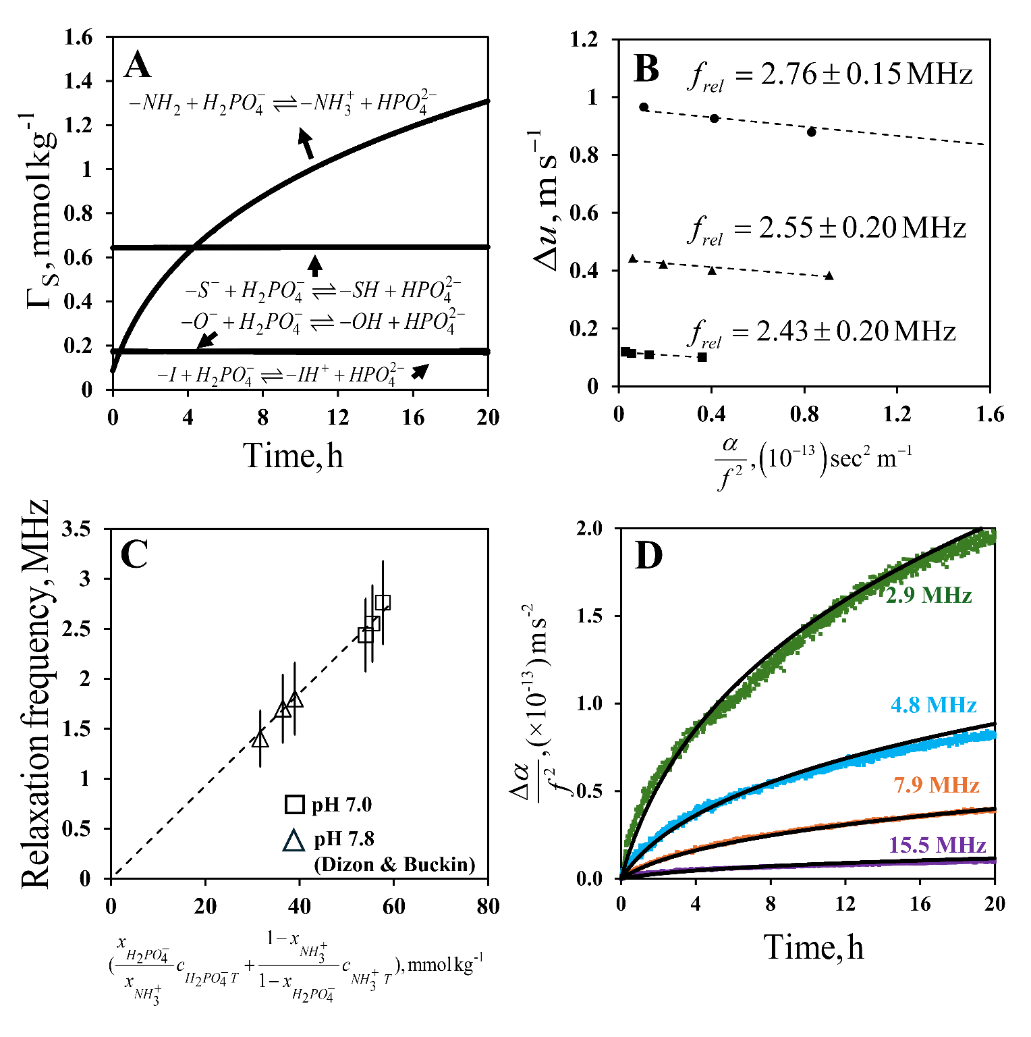


Figure S3 (A) Ultrasonic relaxation analysis of α–chymotrypsin hydrolysis of β–lactoglobulin in 0.1 M phosphate buffer $pH$ 7.0, 25 °C. (A) Evolution of $\Gamma_{S}$ calculated for the relaxation processes present in the hydrolysis mixture. (B) Correlation between the change in ultrasonic velocity $\Delta u$ and ultrasonic attenuation per square of frequency $\Delta\alpha/f^{2}$, measured at four different ultrasonic frequencies (2.9 MHz, 4.8 MHz, 7.9 MHz and 15.5 MHz) at selected hydrolysis time 1300^th^ min (circle symbols); 300^th^ min (triangle symbols) and 50^th^ min (square symbols). Solid lines represent linear fits used to determine $f_{rel}$ (Equation S5, $R^{2}\geq0.9$). (C) Dependence of relaxation frequency on the concentration factor $\left(c_{H_{2}PO_{4}^{-}T}\left(x_{H_{2}PO_{4}^{-}}/x_{NH_{3}^{+}}\right)+c_{NH_{3}^{+}T}\left(1-x_{NH_{3}^{+}}/1-x_{H_{2}PO_{4}^{-}}\right)\right)$, in comparison with $pH$ 7.8 (data taken from (Dizon & Buckin, 2025)). Solid lines represent fit used to determine the $k_{f}^{0}$ (Equation S6, $R^{2}\geq0.99$). (D) Time-dependent changes in $\Delta\alpha/f^{2}$ measured at 2.9 MHz, 4.8 MHz, 7.9 MHz and 15.5 MHz, where the symbols represent the experimental data and the solid lines represent the model fit to determine $\Delta V_{S}$ (Equation S7, $R^{2}\geq0.9$). Parameter uncertainties are reported as 95% confidence intervals.

Figure S4 Baseline-corrected circular dichroism (CD) spectra of β‒lactoglobulin before and during enzymatic hydrolysis by α‒chymotrypsin in 0.1 M phosphate buffer ($pH$ 7.8). Each spectrum was recorded at a specific time point throughout the hydrolysis reaction to monitor structural changes over time.


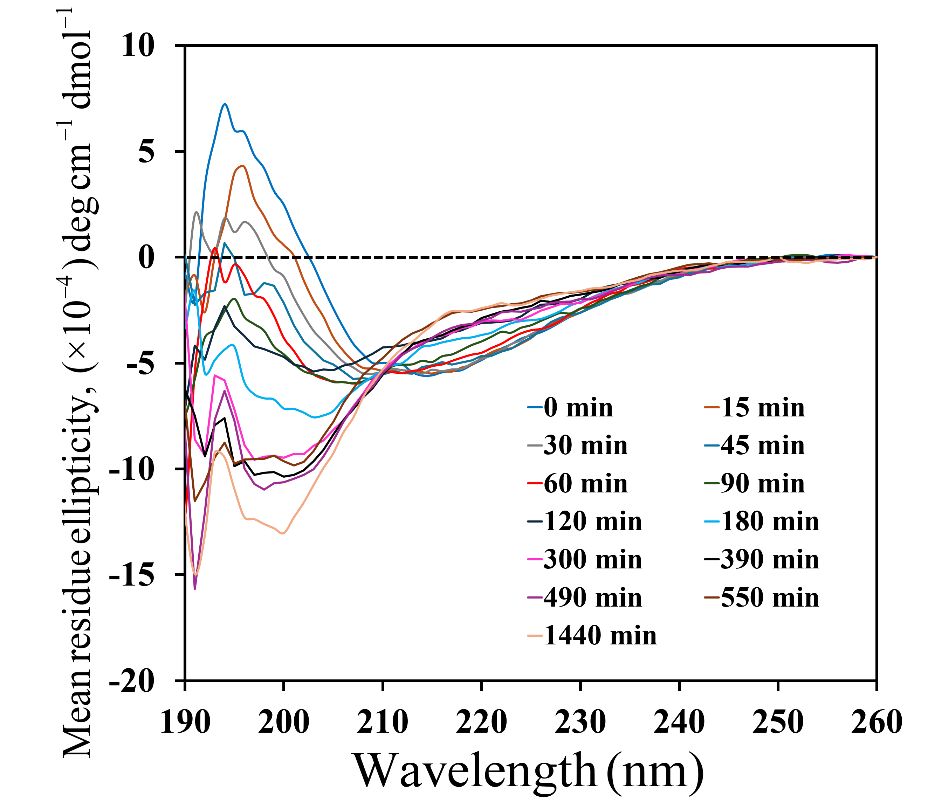


Figure S5 Intrinsic fluorescence emission spectra (excitation at 295 nm) of native β‒lactoglobulin and its hydrolysates recorded at various time points during enzymatic hydrolysis at $pH$ 7.8.


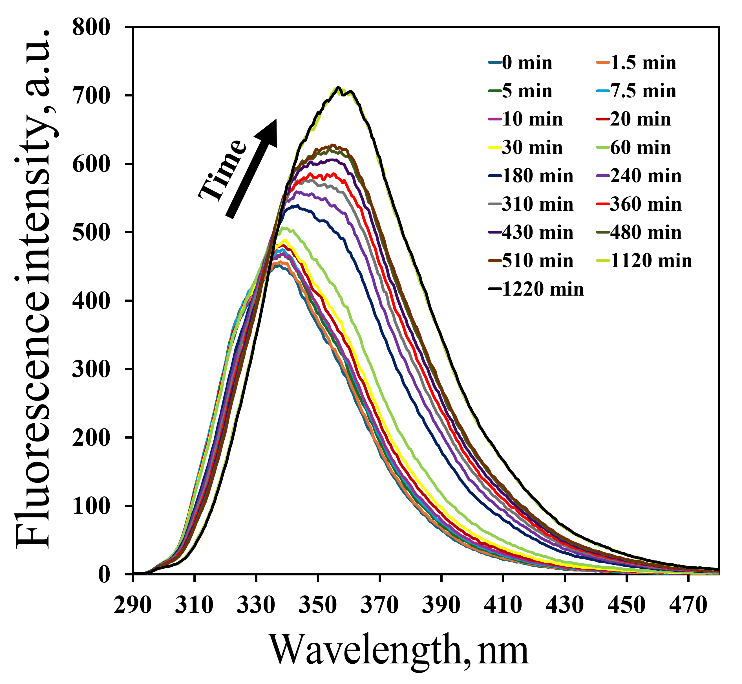

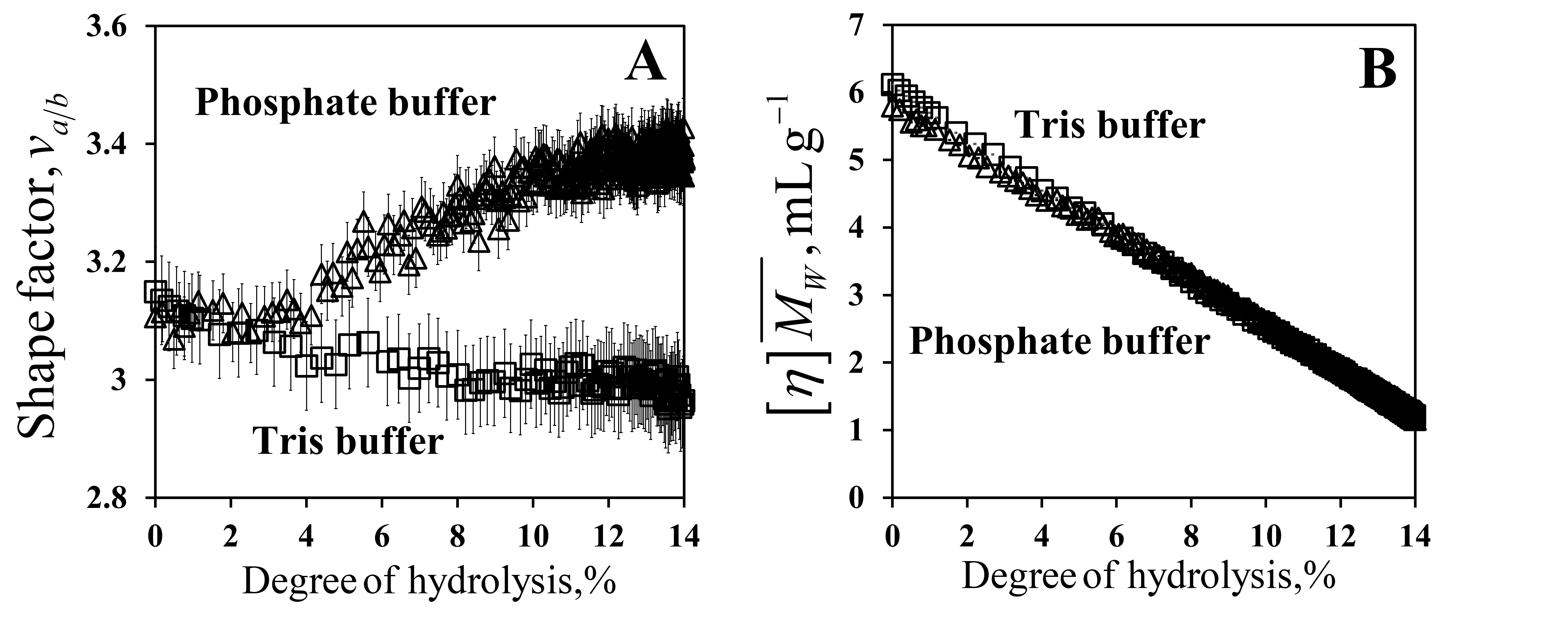


Figure S6 Evolution of (A) universal shape factor $v_{a/b}$ (dimensionless derived from the ratio $\frac{\left[\eta\right]}{v}$) and (B) $\left[\eta\right]\overline{M_{W}}$ (Equation ) for β–lactoglobulin during α–chymotrypsin hydrolysis in 0.1 M phosphate (open triangles) and 0.1 M Tris buffers (open squares), $pH$ 7.8, at 25 °C.

1. Determination of specific volume and adiabatic compressibility during protein hydrolysis

The molar adiabatic compressibility $K_{S}$ can be calculated from the molar volume of the solute $V$ and the concentration increment of the square of ultrasonic velocity $a_{i^{2}}$($\equiv\left(u^{2}-u_{0}^{2}\right)/\left(2u_{0}^{2}c\right)$ (Buckin, 2012)) as:

$$K_{S}=k_{S 0}(2\frac{V}{v_{0}}-2a_{i^{2}}-M_{s})+k_{S 0}c\frac{(\frac{V}{v_{0}}-2a_{i^{2}}-M_{s})^{2}}{1+2ca_{i^{2}}}$$

where $v_{0}$ and $k_{S 0}$ are the specific volume and the specific adiabatic compressibility of pure solvent (water). The parameter $a_{i^{2}}$ is related to the molar concentration increment of ultrasonic velocity $a_{i}$ as $a_{i^{2}}=(1+a_{i}c/2)a_{i}$ (with the replacement of specific parameters by the molar ones), where $a_{i}=\left(u-u_{0}\right)/u_{0}c$. In a non-concentrated system, $a_{i}\equiv a_{i}^{2}$ can be assumed and the second term vanishes to zero:

$$K_{S}=k_{S 0}(2\frac{V}{v_{0}}-2a_{i}-M_{s})$$

Rearrangement of Equation and differencing yield the change in the concentration increment of ultrasonic velocity with respect to the change in molar volume $\Delta V$ and adiabatic compressibility, $\Delta K_{S}$:

$$\Delta a_{i}=\frac{\Delta V}{v_{0}}-\frac{1}{2}\frac{\Delta K_{S}}{k_{S0}}$$

1. Ultrasonic relaxation analyses

The frequency dependence observed in the ultrasonic parameters (Figure S1) during protein hydrolysis is predominantly governed by a relaxation process arising from proton exchange between the N-terminal amino groups ($R-NH_{3}^{+}$) of the hydrolysates and phosphate ions (Buckin & Altas, 2017; Dizon & Buckin, 2025):

$R-NH_{2}+H_{2}PO_{4}^{-}\rightleftarrows R-NH_{3}^{+} + HPO_{4}^{2-}$ (Reaction A)

All species participating in this equilibrium are present at appreciable concentrations under the experimental conditions. Additional proton-transfer reactions involving amino acid side chains such as cysteine ($-SH$group), tyrosine ($-OH$) and histidine ($-I$), which possess $pK_{A}^{app}$ values near neutral $pH$, may also contribute to the observed relaxation:

$\begin{matrix} -S^{-}+H_{2}PO_{4}^{-}\rightleftharpoons-SH+HPO_{4}^{2-} \\ -O^{-}+H_{2}PO_{4}^{-}\rightleftharpoons-OH+HPO_{4}^{2-} \\ -I+H_{2}PO_{4}^{-}\rightleftharpoons-IH^{+}+HPO_{4}^{2-} \end{matrix}$ (Reaction B)

The magnitude of each relaxation process is characterised by its reaction capacity$\Gamma_{S}=\left(\sum_{i=1}^{k+l} y_{i}^{2}/c_{i}\right)^{-1}$, where $y_{i}$ and $c_{i}$ are the stoichiometric coefficient and concentration of component $i$ of the reactions, respectively. The relaxation frequencies of these reactions are expected to fall within a similar range. However, because the concentrations of titratable side chains are lower than the increasing concentration of N-terminal amino groups (depicted in terms of $\Gamma_{S}$ in Figure S3A), their contribution is small and only weakly coupled to the dominant phosphate–N-terminal proton exchange (Dizon & Buckin, 2025). In addition, the high phosphate concentration (0.1 M), which greatly exceeds the protein concentration, further suppresses the coupling effects. Accordingly, the relaxation contributions to ultrasonic velocity and attenuation were approximated by considering only the proton exchange reaction between phosphate ions and $R-NH_{3}^{+}$. The expression of the $\Gamma_{S}$ term can be simplified as:

$$\Gamma_{S}=\left(\frac{1}{c_{H_{2}PO_{4}^{-}T}x_{H_{2}PO_{4}^{-}}\left(1-x_{H_{2}PO_{4}^{-}}\right)}+\frac{1}{c_{NH_{3}^{+}T}\left(1-x_{NH_{3}^{+}}\right)x_{NH_{3}^{+}}}\right)^{-1}$$

The time dependence of ultrasonic attenuation is expected to follow the evolution of $\Gamma_{S}\left(t\right)$.

Following this, the kinetic characterization of the relaxation process can be performed by correlating measured $\Delta u$with $\Delta\alpha/f$ at the measured ultrasonic frequencies and three selected reaction times: 50^th^ min ($c_{bh}$ = 1.0 mmol kg^-1^), 300^th^ min ($c_{bh}$ = 4.0 mmol kg^-1^) and 1300^th^ min ($c_{bh}$ = 8.7 mmol kg^-1^). As shown in Figure S3B, a linear correlation was observed, consistent with the equation:

$$\Delta u=\Delta u_{\infty}-f_{rel}\frac{u^{2}}{2\pi}\left(1+\psi_{ua}\right)\frac{\Delta\alpha}{f^{2}}$$

where $\Delta u_{\infty}$ is the change in ultrasonic velocity at the high-frequency limit ($f\gg f_{rel}$), in the absence of the relaxation effect, and represents the Y-axis intercept. The correction term $\psi_{ua}$ (=$\left(1-\Delta f_{rel}/f_{rel}\right)\left(\left(\Gamma_{S}^{0}\Delta f_{rel}\right)/\left(\Delta\Gamma_{S}f_{rel}\right)\right)/\left(1+\left(\Gamma_{S}^{0}\Delta f_{rel}\right)/\left(\Delta\Gamma_{S}f_{rel}\right)\right)$), which accounts for concentration-dependent effects, was calculated to be very low, varies from 0.005 to 0.06, at $pH$ 7.0. Following this, the effective reaction rate constant $k_{f}^{0}$ was extracted from the linear dependence between $f_{rel}$ and concentration factor $\left(c_{H_{2}PO_{4}^{-}T}\left(x_{H_{2}PO_{4}^{-}}/x_{NH_{3}^{+}}\right)+c_{NH_{3}^{+}T}\left(1-x_{NH_{3}^{+}}/1-x_{H_{2}PO_{4}^{-}}\right)\right)$ (Figure S3C):

$$f_{rel}=k_{f}^{0}\frac{\gamma_{H_{2}PO_{4}^{-}}\gamma_{NH_{2}}}{\gamma_{\left\{T\right\}}}\frac{\rho}{2\pi}\left(\frac{x_{H_{2}PO_{4}^{-}}}{x_{NH_{3}^{+}}}c_{H_{2}PO_{4}^{-}T}+\frac{1-x_{NH_{3}^{+}}}{1-x_{H_{2}PO_{4}^{-}}}c_{NH_{3}^{+}T}\right)$$

where $\frac{\gamma_{H_{2}PO_{4}^{-}}\gamma_{NH_{2}}}{\gamma_{\left\{T\right\}}}$ (where $\gamma_{H_{2}PO_{4}^{-}}$, $\gamma_{NH_{2}}$ and $\gamma_{\left\{T\right\}}$ are the coefficient of activity of phosphate ion $H_{2}PO_{4}^{-}$, protein hydrolysate with N-terminal group $-NH_{2}$ and the transition complex $\left\{T\right\}\approx\left\{-NH_{2}\cdots H_{2}PO_{4}^{-}\right\}$) has the range of values 1.6 – 4.5, with average of 3 for all whey proteins (Dizon & Buckin, 2025). The slight discrepancy can be attributed to variations in the activity coefficients $\gamma_{NH_{2}}$ arising from differences in hydrolysate composition and charge states, which lead to multiple, overlapping relaxation frequencies, broadening the spectral response around $f_{rel}$.

Lastly, the adiabatic reaction volume $\Delta V_{S}$ was determined by fitting the time profiles of $\Delta\alpha/f$ at the measured ultrasonic frequencies, following the fitting algorithm described previously, involving the following equation (Figure S3D):

$$\frac{\Delta\alpha}{f^{2}}\left(t\right)=\pi u\rho^{2}\frac{\Delta V_{S}^{2}}{RT}\Delta\Gamma_{S}\left(t\right)\frac{f_{rel}\left(t\right)}{f_{rel}\left(t\right)^{2}+f\left(t\right)^{2}}\left(1+\frac{\Gamma_{S}^{0}\Delta f_{rel}\left(t\right)}{\Delta\Gamma_{S}\left(t\right)f_{rel}\left(t\right)}\right)$$

where $\Delta\Gamma$ and $\Delta f_{rel}$ are the difference between the values at initial time point and at time $t$ of hydrolysis.

1. Circular dichroism spectroscopy data analysis and secondary structure estimates.

Table S2 lists individual results, and Table S3 summarises averages grouped into α‒helix, β‒sheet, β‒turn, and random coil fractions.

Table S2 Secondary structure composition and assignments for native β‒lactoglobulin (Time 0) and β‒lactoglobulin hydrolysate mixtures, obtained from CD spectra (Figure S4) at 25 °C using the CONTINLL, SELCON3, and CDSSTR methods. Structural components are defined as follows: H(r), regular α‒helix; H(d), distorted α‒helix; S(r), regular β‒sheet; S(d), distorted β‒sheet; Trn, β‒turn; Unrd, unordered or random coil.

| Basis set | 7 |  |  |  |  |  |  |  |
| --- | --- | --- | --- | --- | --- | --- | --- | --- |
| Time 0 min |  |  |  |  |  |  |  |  |
| Method | H(r) | H(d) | S(r) | S(d) | Trn | Unrd |
| SELCON3 | 0.066 | 0.088 | 0.156 | 0.115 | 0.2 | 0.351 |
| CONTINLL | 0.060 | 0.086 | 0.166 | 0.107 | 0.202 | 0.379 |
| CDSSTR | 0.060 | 0.092 | 0.178 | 0.110 | 0.22 | 0.337 |
|  |  |  |  |  |  |  |
| Time 15 min |  |  |  |  |  |  |
| Method | H(r) | H(d) | S(r) | S(d) | Trn | Unrd |
| SELCON3 | 0.052 | 0.078 | 0.161 | 0.091 | 0.181 | 0.409 |
| CONTINLL | 0.031 | 0.032 | 0.208 | 0.107 | 0.205 | 0.418 |
| CDSSTR | 0.061 | 0.093 | 0.152 | 0.100 | 0.210 | 0.390 |
|  |  |  |  |  |  |  |
| Time 30 min |  |  |  |  |  |  |
| Method | H(r) | H(d) | S(r) | S(d) | Trn | Unrd |
| SELCON3 | 0.047 | 0.058 | 0.175 | 0.100 | 0.189 | 0.423 |
| CONTINLL | 0.037 | 0.06 | 0.152 | 0.096 | 0.180 | 0.489 |
| CDSSTR | 0.030 | 0.050 | 0.160 | 0.090 | 0.190 | 0.480 |
|  |  |  |  |  |  |  |
| Time 45 min |  |  |  |  |  |  |
| Method | H(r) | H(d) | S(r) | S(d) | Trn | Unrd |
| SELCON3 | 0.034 | 0.05 | 0.165 | 0.097 | 0.179 | 0.468 |
| CONTINLL | 0.026 | 0.038 | 0.140 | 0.090 | 0.182 | 0.524 |
| CDSSTR | 0.020 | 0.050 | 0.170 | 0.090 | 0.180 | 0.490 |
|  |  |  |  |  |  |  |
| Time 60 min |  |  |  |  |  |  |
| Method | H(r) | H(d) | S(r) | S(d) | Trn | Unrd |
| SELCON3 | 0.033 | 0.049 | 0.124 | 0.119 | 0.147 | 0.524 |
| CONTINLL | 0.036 | 0.059 | 0.123 | 0.125 | 0.170 | 0.485 |
| CDSSTR | 0.033 | 0.050 | 0.132 | 0.126 | 0.170 | 0.485 |
|  |  |  |  |  |  |  |
| Time 90 min |  |  |  |  |  |  |
| Method | H(r) | H(d) | S(r) | S(d) | Trn | Unrd |
| SELCON3 | 0.031 | 0.045 | 0.116 | 0.093 | 0.147 | 0.561 |
| CONTINLL | 0.032 | 0.042 | 0.106 | 0.090 | 0.148 | 0.582 |
| CDSSTR | 0.030 | 0.044 | 0.130 | 0.091 | 0.170 | 0.530 |
|  |  |  |  |  |  |  |
| Time 120 min |  |  |  |  |  |  |
| Method | H(r) | H(d) | S(r) | S(d) | Trn | Unrd |
| SELCON3 | 0.024 | 0.042 | 0.131 | 0.083 | 0.158 | 0.557 |
| CONTINLL | 0.026 | 0.028 | 0.076 | 0.067 | 0.138 | 0.665 |
| CDSSTR | 0.02 | 0.05 | 0.13 | 0.07 | 0.16 | 0.57 |
|  |  |  |  |  |  |  |
| Time 180 min |  |  |  |  |  |  |
| Method | H(r) | H(d) | S(r) | S(d) | Trn | Unrd |
| SELCON3 | 0.031 | 0.036 | 0.088 | 0.067 | 0.131 | 0.638 |
| CONTINLL | 0.025 | 0.028 | 0.086 | 0.066 | 0.137 | 0.658 |
| CDSSTR | 0.020 | 0.040 | 0.120 | 0.070 | 0.140 | 0.600 |
|  |  |  |  |  |  |  |
| Time 300 min |  |  |  |  |  |  |
| Method | H(r) | H(d) | S(r) | S(d) | Trn | Unrd |
| SELCON3 | 0.021 | 0.037 | 0.076 | 0.062 | 0.125 | 0.682 |
| CONTINLL | 0.019 | 0.026 | 0.057 | 0.048 | 0.099 | 0.752 |
| CDSSTR | 0.010 | 0.040 | 0.090 | 0.050 | 0.130 | 0.670 |
|  |  |  |  |  |  |  |
| Time 390 min |  |  |  |  |  |  |
| Method | H(r) | H(d) | S(r) | S(d) | Trn | Unrd |
| SELCON3 | 0.015 | 0.035 | 0.065 | 0.067 | 0.116 | 0.708 |
| CONTINLL | 0.015 | 0.023 | 0.066 | 0.053 | 0.11 | 0.734 |
| CDSSTR | 0.020 | 0.040 | 0.067 | 0.06 | 0.11 | 0.71 |
|  |  |  |  |  |  |  |
| Time 484 min |  |  |  |  |  |  |
| Method | H(r) | H(d) | S(r) | S(d) | Trn | Unrd |
| SELCON3 | 0.017 | 0.023 | 0.008 | 0.038 | 0.087 | 0.829 |
| CONTINLL | 0.011 | 0.021 | 0.046 | 0.039 | 0.076 | 0.811 |
| CDSSTR | 0.010 | 0.033 | 0.180 | 0.060 | 0.107 | 0.630 |
|  |  |  |  |  |  |  |
| Time 540 min |  |  |  |  |  |  |
| Method | H(r) | H(d) | S(r) | S(d) | Trn | Unrd |
| SELCON3 | 0.005 | 0.025 | 0.047 | 0.063 | 0.111 | 0.746 |
| CONTINLL | 0.010 | 0.024 | 0.048 | 0.046 | 0.104 | 0.759 |
| CDSSTR | 0.010 | 0.040 | 0.045 | 0.050 | 0.110 | 0.740 |
|  |  |  |  |  |  |  |
| Time 1440 min | |  |  |  |  |  |
| Method | H(r) | H(d) | S(r) | S(d) | Trn | Unrd |
| SELCON3 | 0.007 | 0.021 | 0.071 | 0.053 | 0.092 | 0.759 |
| CONTINLL | 0.009 | 0.018 | 0.029 | 0.038 | 0.089 | 0.822 |
| CDSSTR | 0.010 | 0.022 | 0.070 | 0.050 | 0.105 | 0.750 |
|  |  |  |  |  |  |  |
| Time 1620 min | |  |  |  |  |  |
| Method | H(r) | H(d) | S(r) | S(d) | Trn | Unrd |
| SELCON3 | 0.003 | 0.015 | 0.045 | 0.037 | 0.083 | 0.821 |
| CONTINLL | 0.006 | 0.023 | 0.058 | 0.041 | 0.082 | 0.797 |
| CDSSTR | 0.003 | 0.03 | 0.056 | 0.038 | 0.088 | 0.790 |

Table S3 Averaged secondary structure contents (α‒helix, β‒sheet, β‒turn, and random coil) at different hydrolysis time points, corresponding to the measured degree of hydrolysis ($d_{h},\%$). Values were derived by averaging the outputs from the CONTINLL, SELCON3, and CDSSTR algorithms shown in Table S2.

| Time, min | $d_{h},\%$^a^ | α‒helix | β‒sheet | β‒turn | random coil |
| --- | --- | --- | --- | --- | --- |
| 0 | 0.0 | 0.151 | 0.277 | 0.207 | 0.356 |
| 15 | 1.99 | 0.116 | 0.273 | 0.199 | 0.406 |
| 30 | 3.25 | 0.094 | 0.258 | 0.186 | 0.464 |
| 60 | 5.09 | 0.087 | 0.249 | 0.162 | 0.498 |
| 90 | 6.46 | 0.074 | 0.209 | 0.155 | 0.558 |
| 120 | 7.50 | 0.063 | 0.186 | 0.152 | 0.597 |
| 180 | 8.97 | 0.060 | 0.166 | 0.136 | 0.632 |
| 300 | 10.72 | 0.051 | 0.128 | 0.118 | 0.701 |
| 390 | 11.54 | 0.049 | 0.126 | 0.112 | 0.717 |
| 484 | 12.25 | 0.038 | 0.124 | 0.090 | 0.757 |
| 540 | 12.57 | 0.038 | 0.100 | 0.108 | 0.748 |
| 1440 | 14.50 | 0.029 | 0.104 | 0.095 | 0.777 |
| 1620 | 15.00 | 0.027 | 0.092 | 0.084 | 0.803 |

^a^Degree of hydrolysis $d_{h},\%$ is calculated as the ratio $\frac{c_{bh}\left(t\right)}{c_{b}^{0}}$ (Equation 6 in main text); where $c_{bh}\left(t\right)$ is the concentration of peptide bonds hydrolysed during hydrolysis and $c_{b}^{0}$ is the initial concentration of hydrolysable peptide bonds in the protein substrate.

1. Intrinsic tryptophan fluorescence analysis

Table S4 Fitted maximum fluorescence emission wavelength $\lambda_{\max}$ within the selected fitting window $\Delta\lambda_{fitting}$, and the corresponding calculated degree of demasking $1-x\left(t\right)$ at various reaction extent $\xi$ during the hydrolysis reaction.

| Time, min | $\xi$,% ^a^ | $\Delta\lambda_{fitting}$, nm^b^ | $\lambda_{\max}=\frac{-b}{\left(2a\right)}$, nm^c^ | Degree of demasking,$1-x\left(t\right)$^d^ |
| --- | --- | --- | --- | --- |
| 0 | 0 | 30 | 337.24 | 0 |
| 1.5 | 0.7 | 30 | 337.42 | 0.008 |
| 5 | 3.5 | 30 | 337.61 | 0.015 |
| 7.5 | 4.4 | 30 | 337.945 | 0.029 |
| 10 | 7.2 | 30 | 338.33 | 0.045 |
| 20 | 11.5 | 30 | 338.89 | 0.068 |
| 30 | 15.2 | 30 | 339.55 | 0.095 |
| 60 | 23.0 | 30 | 341.11 | 0.158 |
| 180 | 40.2 | 30 | 347.42 | 0.417 |
| 240 | 44.7 | 30 | 349.38 | 0.497 |
| 310 | 48.3 | 50 | 350.62 | 0.548 |
| 360 | 50.8 | 50 | 351.67 | 0.591 |
| 430 | 52.9 | 50 | 352.495 | 0.624 |
| 480 | 54.6 | 50 | 353.16 | 0.652 |
| 510 | 55.5 | 50 | 353.51 | 0.666 |
| 1120 | 63.4 | 50 | 357.03 | 0.810 |
| 1220 | 63.8 | 50 | 357.14 | 0.815 |

^a^calculated as $\xi,\%=\left(d_{h}/0.2236\right)\times100$ ^b^fitting window around the $\lambda_{\max}$ ^c^determined from the fitting equation $a\lambda^{2}+b\lambda+c$ ^d^calculated as $x\left(t\right)=\left(\lambda_{\max}\left(t\right)-\lambda_{\max}^{0}\right)/\left(\lambda_{\max}^{\infty}-\lambda_{\max}^{0}\right)$ (Equation 13 in main text), where $\lambda_{\max}^{\infty}$= 361.7 nm.

1. Light scattering analysis

Apparent weight-average molecular mass $\overline{M_{W}}$ of the unhydrolyzed protein and its hydrolysate mixtures was calculated using the light scattering data according to Rayleigh Equation:

$$\frac{KC}{R_{\theta}}=\frac{1}{\overline{M_{w}}P(\theta)}+2A_{2}C$$

where $C$ is the protein concentration (g cm^–3^ unit), $P(\theta)$ is the angular scattering factor, $R_{\theta}$ is the Rayleigh ratio of the scattered to incident light, $A_{2}$ is the second virial coefficient, and $K$ is the optical constant:

$$K=\frac{4\pi^{2}n_{0}^{2}(dn/dC)^{2}}{N_{A}\lambda_{0}^{4}}$$

where $n_{0}$ is the refractive index of the sample solvent (water, in this case, with $n_{0}$ = 1.3369 at 25 °C, derived from a temperature dependence function (Harvey et al., 1998): $5.9169\times10^{-9}T^{3}-6.892\times10^{-6}T^{2}+2.4268\times10^{-3}T+1.0692$, where temperature is in Kelvin unit); $\frac{dn}{dC}$ (= 0.175 (Barer & Joseph, 1954)) is the specific refractive increment of protein; $N_{A}$ = 6.022 x 10^23^ mol^-1^ is the Avogadro’s number; and $\lambda_{0}$ = 488 nm is the incident laser wavelength. The Rayleigh ratio $R_{\theta}$ was calculated as:

$$R_{\theta}=\frac{I_{P}n_{0}^{2}}{I_{toluene}n_{toluene}^{2}}R_{toluene}$$

where $I_{P}$ and $I_{toluene}$ are the light intensity of the protein sample and toluene, respectively; $n_{toluene}$ (= 1.5068) is the refractive index of toluene, derived from its incident wavelength dependence as (Rubio et al., 2004): $n_{toluene}= -1.504\times10^{-9}\lambda_{0}^{3}+2.833\times10^{-6}\lambda_{0}^{2}-1.845\lambda_{0}+1.905$), and $R_{toluene}$ (= 3.05 x 10^-5^ cm^-1^), is the Rayleigh ratio of toluene, derived from its incident wavelength dependence as (Wu, 2010): $R_{toluene}= 4.90\text{ }x\text{ }10^{6} \lambda_{0}^{-4.17} \left(\text{cm}^{-1}\right)$ at 25 °C.

1. Hydrodynamic analysis

Macromolecular dimensions in solution are related to intrinsic viscosity $\left[\eta\right]$ (de la Torre et al., 1997; Harding, 1997; Yang, 1962). The dimensionless molecular shape parameter$v_{a/b}$ (with respect to aspect ratio $a/b$) was calculated as:

$$v_{a/b}=\frac{\left[\eta\right]}{v}$$

where $v$ is the specific volume. This quantity represents a universal viscosity function that depends on molecular asymmetry and includes contributions from bound solvent. The hydrated molecular volume $V_{M}$ was computed as:

$$V_{M}=\frac{\left[\eta\right]M_{W}}{v_{a/b}N_{A}}$$

This expression follows from classical hydrodynamic theory relating intrinsic viscosity to effective particle volume under the assumption of equivalent hydrodynamic spheres, an approximation appropriate for native and early-stage β‒lactoglobulin. Additionally, the product $\left[\eta\right]\overline{M_{W}}$ is related to the hydrodynamic radius $R_{H}$:

$$R_{H}=\left(\frac{4\left[\eta\right]\overline{M_{W}}}{3\pi v_{a/b}N_{A}}\right)^{\frac{1}{3}}$$

which is equivalent to $R_{H}=\left(V_{M}4/3\pi\right)^{\frac{1}{3}}$. These estimates assume isotropic hydrodynamic equivalence and do not explicitly account for segmental flexibility. However, this becomes less representative as hydrolysis generates shorter, more flexible peptides.

Lastly, the quantity that is usually employed to express volume hydration of biopolymer is the hydration ratio $\delta$ of the grams of water per gram of biomacromolecule, which can be calculated from the density data through the following equation:

$$\delta=\frac{\left(v-\overline{v}\right)}{v_{0}}$$

where $\overline{v}$ is the partial specific volume. The hydration expansion factor $h$ is then calculated using:

$$h=1+\delta\frac{v}{\overline{v}}$$

This treatment assumes additive volumes and uniform solvent association.

**References**

Barer, R., & Joseph, S. (1954). Refractometry of Living Cells Part I. Basic Principles. *Journal of Cell Science*, *s3-95*(32), 399–423. <https://doi.org/10.1242/jcs.s3-95.32.399>

Buckin, V. (2012). Application of High-Resolution Ultrasonic Spectroscopy for analysis of complex formulations. Compressibility of solutes and solute particles in liquid mixtures. *IOP Conference Series: Materials Science and Engineering*, *42*(1), 012001. <https://doi.org/10.1088/1757-899X/42/1/012001>

Buckin, V., & Altas, M. C. (2017). Ultrasonic Monitoring of Biocatalysis in Solutions and Complex Dispersions. *Catalysts*, *7*(11), 336. <https://doi.org/10.3390/catal7110336>

de la Torre, J. G., Carrasco, B., & Harding, S. E. (1997). SOLPRO: Theory and Computer Program for the Prediction of SOLution PROperties of Rigid Macromolecules and Bioparticles. *European Biophysics Journal*, *25*(5), 361–372. <https://doi.org/10.1007/s002490050049>

Dizon, M., & Buckin, V. (2023). Ultrasonic Monitoring of Enzymatic Hydrolysis of Proteins. 1. Effects of Ionisation. *Food Hydrocolloids*, *144*, 108866. <https://doi.org/10.1016/j.foodhyd.2023.108866>

Dizon, M., & Buckin, V. (2025). Ultrasonic Monitoring of Enzymatic Hydrolysis of Proteins. 2. Relaxation Effects. *Food Hydrocolloids*, *158*, 110221. <https://doi.org/10.1016/j.foodhyd.2024.110221>

Harding, S. E. (1997). The Intrinsic Viscosity of Biological Macromolecules. Progress in Measurement, Interpretation and Application to Structure in Dilute Solution. *Progress in Biophysics and Molecular Biology*, *68*(2), 207–262. <https://doi.org/10.1016/S0079-6107(97)00027-8>

Harvey, A. H., Gallagher, J. S., & Sengers, J. M. H. L. (1998). Revised Formulation for the Refractive Index of Water and Steam as a Function of Wavelength, Temperature and Density. *Journal of Physical and Chemical Reference Data*, *27*(4), 761–774. <https://doi.org/10.1063/1.556029>

Rubio, J. E. F., Arsuaga, J. M., Taravillo, M., Baonza, V. n. G., & Cáceres, M. (2004). Refractive Index of Benzene and Methyl Derivatives: Temperature and Wavelength Dependencies. *Experimental Thermal and Fluid Science*, *28*(8), 887–891. <https://doi.org/10.1016/j.expthermflusci.2004.01.008>

Wu, H. (2010). Correlations Between the Rayleigh Ratio and the Wavelength for Toluene and Benzene. *Chemical Physics*, *367*(1), 44–47. <https://doi.org/10.1016/j.chemphys.2009.10.019>

Yang, J. T. (1962). The Viscosity Of Macromolecules In Relation To Molecular Conformation. In C. B. Anfinsen, M. L. Anson, K. Bailey, & J. T. Edsall (Eds.), *Advances in Protein Chemistry* (Vol. 16, pp. 323–400). Academic Press. <https://doi.org/10.1016/S0065-3233(08)60032-7>
